# Modifiable Risk Factors for Alzheimer’s Disease

**DOI:** 10.3389/fnagi.2019.00146

**Published:** 2019-06-24

**Authors:** George A. Edwards III, Nazaret Gamez, Gabriel Escobedo Jr., Olivia Calderon, Ines Moreno-Gonzalez

**Affiliations:** ^1^The Mitchell Center for Alzheimer’s Disease and Related Brain Disorders, Department of Neurology, The University of Texas Houston Health Science Center at Houston, Houston, TX, United States; ^2^Networking Research Center on Neurodegenerative Diseases (CIBERNED), Department of Cell Biology, Facultad Ciencias, Universidad de Malaga, Malaga, Spain

**Keywords:** Alzheimer’s disease, risk factors, comorbidities, vascular disease, traumatic brain injury, epilepsy, depression, lifestyle

## Abstract

Since first described in the early 1900s, Alzheimer’s disease (AD) has risen exponentially in prevalence and concern. Research still drives to understand the etiology and pathogenesis of this disease and what risk factors can attribute to AD. With a majority of AD cases being of sporadic origin, the increasing exponential growth of an aged population and a lack of treatment, it is imperative to discover an easy accessible preventative method for AD. Some risk factors can increase the propensity of AD such as aging, sex, and genetics. Moreover, there are also modifiable risk factors—in terms of treatable medical conditions and lifestyle choices—that play a role in developing AD. These risk factors have their own biological mechanisms that may contribute to AD etiology and pathological consequences. In this review article, we will discuss modifiable risk factors and discuss the current literature of how each of these factors interplay into AD development and progression and if strategically analyzed and treated, could aid in protection against this neurodegenerative disease.

## Introduction

Alzheimer’s dementia is an age-related neurodegenerative disease characterized by several neuropathological markers including extracellular amyloid-β (Aβ) plaques, intracellular neurofibrillary tangles (NFTs), inflammation, synaptic impairment, and neuronal loss that leads to cognitive impairment (Querfurth and LaFerla, 2010). A multitude of studies has shown strong evidence for the concept that the misfolding, aggregation and brain accumulation of protein aggregates are a triggering event in the pathogenesis of Alzheimer’s disease (AD) and responsible for the subsequent pathological alterations that lead to the clinical disease (Moreno-Gonzalez and Soto, 2011). Due to the progressive aging of the population, the number of people affected by AD in the United States is predicted to reach 14 million by the year 2050 (Mebane-Sims and Alzheimer’s Association, 2009). Nowadays, there is not a definitive cure for this disease. Treatment and daily care of AD patients are considered costly in emotional and economical aspects. Available drugs used to treat AD are expensive, and they focus only to alleviate symptoms that are invariably fatal. The etiology of sporadic AD—more than 95% of cases—is not completely understood. The lack of knowledge about sporadic AD etiology makes it very difficult to prevent its onset and detect risk factors. Although the avoidance or prevention of modifiable risk factors may not have full impact in the future development of the disease, a recent study determined that good lifestyle habits and management of comorbidities may lead to a lower risk of dementia (Baumgart et al., 2015). Therefore, identifying potential risk factors may facilitate a reduction in the burden of people affected by AD. In this review article, we will discuss proposed modifiable risk factors for AD, focusing on their effect on protein aggregation and deposition, and whether their prevention and control may have a direct impact in the potential to develop this dementia.

## Comorbidities

### Vascular Diseases

The cerebrovascular network and the neurovascular control mechanisms have a pivotal role in maintaining the activity and integrity of the brain by assuring constant blood flow (Iadecola, 2004). In this process, the neurovascular unit—a specialized entity of neurons, astrocytes and vascular endothelial cells—has a crucial function. Alterations in this vascular system contribute to a reduction in global cerebral perfusion leading to brain dysfunction and cognitive impairment (Iadecola, 2013), thereby introducing the concept of the vascular hypothesis of AD. In this hypothesis, vascular pathologies promote the neuropathologic hallmarks of AD (de la Torre, 2018). Indeed, vascular risk factors are critically involved in the progression of dementia leading the conversion from mild cognitive impairment (MCI) to AD (Luchsinger et al., 2005; Li et al., 2011). Less than 10% of demented individuals develop only vascular dementia (Brenowitz et al., 2017). There is a growing body of evidence that supports the idea of vascular factors as contributors of the pathological mechanisms of AD. Epidemiologically, different risk factors for vascular diseases have been shown as significant risk factors for AD (Panpalli Ates et al., 2016). It has been suggested that the link between cerebrovascular disease (CVD) and AD is even much more important than the influence of aging (Love and Miners, 2016). Some of these common risk factors shared between CVD and AD are hypertension, diabetes, atrial fibrillation, atherosclerosis, hypercholesterolemia, and apolipoprotein E (ApoE) genotype (Vijayan and Reddy, 2016).

A history of prehypertension and hypertension in midlife or late in life increases the risk of developing dementia and enhances the neuropathology of AD (Dickstein et al., 2010; Gottesman et al., 2017a). The Honolulu-Asia study revealed that hypertensive patients showed an abundance of amyloid plaques and NFTs in the brain and atrophied hippocampus (Launer et al., 2000). Furthermore, high blood pressure promotes atherosclerosis in cerebral arteries, blocking the cerebral blood supply (Ninomiya et al., 2011), leading to lacunar or cortical infarcts, and, ultimately, cognitive impairment (Dickstein et al., 2010). Angiotensin-converting enzyme (ACE), that regulates blood pressure, is able to degrade Aβ (Hemming and Selkoe, 2005) and the use of anti-hypertensive medications, such as ACE inhibitors, to reduce the risk to develop AD may in fact lead to an opposite effect than desired. However, ACE inhibitors have been proven to not increase amyloid burden *in vivo* (Hemming et al., 2007). In humans, ACE inhibitors do not have a beneficial effect in cognitive impairment either (Peters et al., 2008), but other antihypertensive treatments may still be helpful to reduce the risk of AD. Hence, hypertension could be established as one of the strongest risk factors for AD. On the other hand, it has been proposed that hypertension could be induced by the action of Aβ before dementia onset—being responsible for high blood pressure and cerebrovascular impairment (Petrovitch et al., 2000). Therefore, hypertension could be just a result of Aβ accumulation rather than a risk or a combination of both.

Hypertension is the main risk factor for stroke, a phenomenon that deprives the supply of blood flow to the brain. In fact, the severity of stroke is higher in diabetic patients, which increases the rate of death (Air and Kissela, 2007). Clinical history of stroke is associated with a prevalence of dementia, denoted as post-stroke dementia (Pendlebury and Rothwell, 2009), doubling the risk of developing AD in the elderly (Sun et al., 2006). Among single or multiple stroke patients, post-stroke dementia is a common outcome. Mechanistically, there are several processes that potentially link AD and stroke. It has been proposed that stroke could promote Aβ production, hamper Aβ clearance, and/or aggravate synaptic and neuronal loss already triggered by Aβ and tau pathology (Sun et al., 2008; Garcia-Alloza et al., 2011; Hongpaisan et al., 2011).

Heart disease (atrial fibrillation, arrhythmias, or cardiac arrest) causes a reduction in cerebral perfusion, leading to nerve cell damage (Kwok et al., 2011), brain dysfunction, and cognitive decline (Alosco et al., 2013). Atrial fibrillation is known as another risk factor for stroke, increasing the prevalence of AD and dementia (Ott et al., 1997; Kilander et al., 1998). The association between heart failure and cognitive impairment is supported by the induction of brain hypoxia and neuronal loss after a hypoperfusion event (Muqtadar et al., 2012). In addition, an elevation in Aβ42 serum levels has been reported following a cardiac arrest episode, which would also contribute to AD neuropathology (Zetterberg et al., 2011). Overall, cardiovascular diseases seem to induce a lack of perfusion/oxygenation in the brain, leading to cognitive impairment and dementia mediated by an increase in Aβ levels due to different mechanisms. Although already existing Aβ aggregates can also induce cerebral perfusion impairment, a history of hypertension, stroke or heart disease can be considered a risk factor to develop AD.

The increased risk of developing AD dementia is also associated with atherosclerosis, a common vessel disorder in the elderly. AD patients show atherosclerosis in the circle of Willis (cerebral arterial circle at the base of the brain) much more severe and more frequently than healthy age-matched controls (Roher et al., 2003), and hypertension can have a role in promoting this intracranial atherosclerosis. This intracranial atherosclerosis reduces the brain blood perfusion and is linked to an increase in neuritic plaque burden and higher Braak stage in AD patients (Beach et al., 2007). Cholesterol has been consistently associated to AD. High levels of cholesterol have been linked to increased Aβ levels and greater cognitive impairment and progression in AD. Cholesterol seems to impair Aβ degradation and promote its production (Barbero-Camps et al., 2018). In fact, the use of statins, a cholesterol-lowering medication, such as simvastatin, has shown to lower the risk of AD diagnosis particularly in women (Zissimopoulos et al., 2017) even in ApoE homozygotes (Geifman et al., 2017) and levels of phospho-tau in the cerebrospinal fluid (CSF; Li G. et al., 2017). The proposed mechanism is the direct interaction of statins and Aβ protofibrils (Shakour et al., 2019), inhibition of apoptosis (Hu et al., 2018). Therefore, hypercholesterolemia has been suggested to be a high-risk factor for AD and cholesterol-lowering medication should be considered as a preventive therapy for dementia.

Cerebral amyloid angiopathy (CAA) is a condition where Aβ deposits accumulate within the walls of the meningeal and intracerebral arteries, arterioles, and very rarely, veins and capillaries. This engenders a thickening of vessels walls and constriction of vascular lumen thereby promoting potential micro-aneurysms. This pathology increases the risk to develop hemorrhages, ischemic lesions, and encephalopathies, resulting in profound cerebral damage that contributes to neurodegeneration and cognitive dysfunction (Ellis et al., 1996; Haglund et al., 2006). CAA is associated to a more rapid cognitive decline in both demented and non-demented persons (Pfeifer et al., 2002). Certainly, CAA has a close association with AD and additive effects on the risk of developing dementia through AD pathology. The diagnosis of probable AD is related to the presence of CAA. In fact, CAA is highly prevalent in AD patients, being present in about 80%–90% of AD patients (Arvanitakis et al., 2011). Moreover, the role of hypertension has a significant additional causal factor that contributes to the progression of CAA-related vasculopathies.

Other CVDs have been described in the aging and AD brain supporting that cerebrovascular dysfunction contributes to neurodegeneration, cognitive dysfunction, and lowers the threshold for developing AD dementia. These cerebrovascular pathologies are cortical infarcts, lacunes, hemorrhages, microbleeds, intracranial small vessel atherosclerosis-arteriosclerosis, and blood brain barrier (BBB) dysfunction (Toledo et al., 2013). Generally, a higher number and the extent of cortical infarcts is directly associated with a higher risk of dementia. Extensive CAA and cerebral small vessel disease have also been proposed to contribute to neurodegeneration in AD (Toledo et al., 2013). Likewise, numerous microbleeds contribute to cognitive function decline and severe white matter lesions that lead to a 4-fold increased risk of developing MCI (Benedictus et al., 2015) that can eventually lead to AD development.

Regarding the effect of CVD on protein misfolding and deposition, there is a greater tendency of amyloid accumulation in patients with vascular risk factors (Langbaum et al., 2012; Gottesman et al., 2017b). Vascular insufficiency results in hypoperfusion and hypoxia that activate the amyloid precursor protein (APP) cleavage enzyme β-secretase (Xu et al., 2007) and facilitates a robust deposition of fibrillar amyloid. Therefore, Aβ not only promotes cerebrovascular dysregulation increasing the brain susceptibility to ischemia but also ischemia upregulates Aβ cleavage and its accumulation. On the other hand, the main Aβ clearance mechanisms are altered and damaged under the presence of vascular dysfunction contributing to parenchymal and vascular accumulation of Aβ (Garcia-Alloza et al., 2011). Tau hyperphosphorylation and NFTs are also associated with vascular risk and the synergistic effect of elevated Aβ burden (Vemuri et al., 2017; Rabin et al., 2019). It has been also described that increased plasma levels of Aβ are linked to vascular disease both in the brain (white matter lesions and microbleeds) and in the periphery (hypertension, diabetes and ischemic heart disease; Janelidze et al., 2016). It should be noted that the contributions of vascular dysfunction occur at the early stages of AD pathophysiology and may represent a casual pathway towards dementia, facilitating an earlier diagnosis of AD. Furthermore, the vascular component is a promising target to decrease the risk of dementia and the neuropathological progression of AD. Nonetheless, further studies are required to elucidate the mechanisms underlying vascular pathologies as they relate to AD and dementia. This, along with the development of precise vascular biomarkers will be fundamental to discover new ways to prevent and treat AD and related dementias. Therefore, the improvement in the vascular health and the control of vascular risk factors may reduce the risk of developing vascular pathologies that trigger AD neuropathology.

### Type 2 Diabetes

Diabetes is estimated to affect over 30.3 million people with over 7.2 million undiagnosed, and 90%–95% of these cases are delineated as type 2 diabetes (T2D; Centers for Disease Control and Prevention, 2017). T2D is a complex metabolic disorder that is characterized prominently by hyperinsulinemia, insulin resistance, glucose metabolism impairments, and, ultimately, pancreatic β-cell destruction. In T2D, pancreatic β-cells secrete excessive insulin in response to insulin resistance causing hyperinsulinemia, while allowing blood glucose levels (BGLs) to be maintained. As this continues over time, it begins to burden the β-cells, leading to insulin insufficiency and finally causing T2D. T2D and AD have a strong epidemiological link—so substantial that some researchers define AD as type 3 diabetes. T2D is proposed to increase the risk of AD and dementia from 1.3 up to 5.5 times and the Rotterdam study in the 1990s described T2D having double the risk for AD and dementia (Ott et al., 1999; Li et al., 2015). T2D patients are at ~60% greater risk for the development of dementia compared with individuals without diabetes (Chatterjee et al., 2016). Additional evidence of a systematic analysis concluded that T2D is convincingly a major risk factor for AD and vascular dementia (Bellou et al., 2017). There are simultaneous influences within each of these diseases that also adjoin T2D and AD, such as progressing age, diet, body mass index (BMI) and obesity, and sedentary lifestyle. In fact, adiposity, being overweight, or obese is a chief cause for insulin resistance (Luchsinger and Gustafson, 2009). There is a strong epidemiological link for T2D and AD and this may be due to their shared pathological mechanisms (Baglietto-Vargas et al., 2016).

Hyperinsulinemia has been associated with AD risk as indicated by such studies as the Honoulu-Asia Aging study (Luchsinger and Gustafson, 2009). Other than regulating the peripheral metabolism, insulin has insulin receptors expressed throughout the central nervous system (CNS). The function of brain insulin receptors is not clearly understood. It is known to regulate circuit function and plasticity by controlling synapse density and plays a role in the cholinergic system. Impairments in insulin receptors and hyperinsulinemia have been associated with aging and AD. A decreased level in insulin receptors and their sensitivity in AD patients compared to middle-aged controls and expression and metabolism of Aβ and tau are also affected (Frölich et al., 1998; Sims-Robinson et al., 2010). Moreover, irregular insulin levels can disrupt the cholinergic system, which is also compromised in AD, as insulin aids in stimulating choline acetyltransferase (ChAT; Rivera et al., 2005). Insulin degrading enzyme (IDE) is vital for the degradation of insulin and Aβ. Thus, hyperinsulinemia can lead to a competition of insulin and Aβ for IDE, thereby increasing amyloid levels in the brain. Loss-of-function mutations of IDE in rodents exhibit glucose intolerance and accruement of Aβ aggregates, whereas, IDE action revealed opposite results (Shen et al., 2006). Downstream insulin signaling pathways are also affected. Pathways that are known to be involved in AD pathogenesis such as mitogen-activated protein kinase (MAPK), protein kinase B (Akt), and glycogen synthase kinase-3β (GSK-3β) are altered due to insulin dysregulation. MAPK expression is correlated with Aβ production and NFTs and is increased in AD patients. Under insulin resistance, Akt signaling can inhibit GSK-3β, which dephosphorylates and activates glycogen synthase in glycogenesis and ultimately resulting in the hyperphosphorylation of tau. Accordingly, the cognitive impairment that is noted in both T2D and AD could be intervened by insulin administration. Tied to insulin issues is the dysfunction of glucose metabolism. The brain is estimated to consume 20% of energy stored in the body, and neurons depend on a steady peripheral transport of glucose through the BBB facilitated by glucose transporters (GLUTs), especially GLUT-1 and -3. Deficiency in GLUT-1 and -3 is reported in AD brains, and this decrease correlated to the decrease in O-GlcNAcylation, hyperphosphorylation of tau, and to the density of NFTs (Liu et al., 2008, 2009). Indeed, imaging techniques such as Fluorodeoxyglucose (FDG)-PET imaging are able to detect glucose metabolism alterations in AD related to anatomical areas associated with pathology and preceding cognitive impairments (Ballard et al., 2011; Nordberg, 2015).

Both AD and T2D are diseases related to protein misfolding and aggregation (Soto, 2003; Moreno-Gonzalez and Soto, 2011; Morales et al., 2013). In T2D, the aggregation of an amyloidogenic protein called islet amyloid polypeptide (IAPP) or amylin is seen in up to 96% of T2D patients in pancreatic β-cells (Clark et al., 1995; Westermark, 2011). IAPP is a hydrophobic hormone co-secreted with insulin into blood circulation at 1:100 ratio. It contributes to glycemic control by slowing down gastric emptying and inhibiting digestive secretion and other pancreatic hormones. Although it is not clear that IAPP is a cause or an effect of T2D, amyloidogenic IAPP aggregates are toxic and have been proposed to destroy β-cells and facilitate the progression of the disease (Westermark, 2011; Abedini et al., 2015). In fact, genetically modified rodent models expressing human IAPP demonstrate aggressive diabetic-like phenotype, such as insulin impairments and hyperglycemia, with IAPP deposits and β-cell loss (Clark et al., 1995; Janson et al., 1996). Interestingly, IAPP has been discovered in AD brains, whereas Aβ and tau have been found in T2D pancreas; in addition, these proteins were seen to co-localize in brain and pancreas (Miklossy et al., 2010; Valente et al., 2010; Moreno-Gonzalez et al., 2017). Moreover, T2D patients display increased amounts of NFTs and Aβ in the hippocampus (Miklossy et al., 2010). In AD patients, there is an extensive prevalence of IAPP compared to non-AD (Janson et al., 2004). Brain of T2D/AD patients show an augmented number of cortical Aβ plaques and tau-positive cells compared to affected AD brains suggesting that T2D/AD patients have a more severe pathology with much more rapid progression (Bretherton-Watt and Bloom, 1991). Therefore, these amyloidogenic proteins may interact directly. IAPP [8–18] and IAPP [22–28] sequences were noted hot regions for IAPP and Aβ40 interaction (Kapurniotu et al., 2010). One proposed mechanism is the cross-seeding of Aβ and IAPP when oligomers composed by one protein seed the aggregation of a different protein by a seeding-nucleation process (Morales et al., 2013; Jucker and Walker, 2015; Moreno-Gonzalez et al., 2017). Many studies reveal a successful heterologous seeding aggregation when both proteins are present *in vitro* (Berhanu et al., 2013; Yan et al., 2014; Moreno-Gonzalez et al., 2017). Double transgenic mice overexpressing both Aβ in brain and IAPP in pancreas show increased Aβ burden compared to controls (Moreno-Gonzalez et al., 2017). IAPP pancreatic homogenate injected into an AD mouse model provided a potent seeding effect by accelerating AD pathology and impairing memory (Moreno-Gonzalez et al., 2017). Independently of the mechanism of action, T2D can be considered one of the main risk factors for AD.

### Traumatic Brain Injury

Recent research has posited that traumatic brain injury (TBI) is a robust factor that leads to the advancement of AD or dementia. The severity of TBI is quantified by the Glascow Coma Scale (GCS). Severe TBI (sTBI) represents head injuries that result in permanent or an extended period of unconsciousness, amnesia, or death following a head injury with a GCS of 3–8. Moderate TBI involves a period of unconsciousness or amnesia from 30 min ≥24 h with a GCS of 9–12. Mild TBI (mTBI) is recognized as head injuries that cause a brief state of altered consciousness resulting in ≤30 min of unconsciousness, though most mTBIs do not result in a loss of consciousness. mTBI represents a majority of reported cases and has been linked to AD pathology (Edwards et al., 2017). The harsher the TBI the greater risk of developing AD (Graves et al., 1990; Guo et al., 2007). World War II veterans that had a TBI event had an increased risk of developing AD (Plassman et al., 2000). After a TBI event, dementia diagnosis was found to be strongest within the first year (4–6 times) but maintained significance up to 30 years (Nordström and Nordström, 2018). In a large cohort study, the overall risk of dementia in individuals with a history of TBI was 24% higher than those without a history of TBI. A sTBI increased AD risk by 35% and a single mTBI or concussion increased the risk by 17%. The risk of dementia was increased with the number of TBI events—33% higher for two or three TBIs, 61% higher for four TBIs, and 183% higher for five or more TBIs (Fann et al., 2018), demonstrating the link between TBI severity and the number of events and dementia. TBI begins with an instant, irreversible initial blow or impact causing direct damage to the surrounding neuronal and astroglial cells and vasculature. Primary insult can either be a focal injury or diffuse axonal injury (DAI). The impact can trigger a rapid necrosis due to the mechanical damage, edema, increased intracranial pressure, and ischemia. This will secondarily lead to neuronal excitotoxicity, mitochondrial dysfunction, oxidative stress, inflammation, synaptic dysfunction, axonal degeneration, neuronal death, and, ultimately, instigating cognitive and behavioral impairments (Gentleman et al., 2004; Breunig et al., 2013). Therefore, TBI shares many of the molecular mechanisms observed in AD. Following TBI, multiple cell death pathways get activated leading to synapsis reduction and finally neuronal loss as loss of total brain volume in the hippocampus, cortex and other medial temporal lobe structures, and elevation in ventricular volume has been described (Blennow et al., 2016). This neurodegenerative process initiated by TBI may trigger the development of memory problems that may then convert into AD.

Numerous *in vivo* studies demonstrate Aβ and tau pathophysiology along with other pathological consequences alike to AD following TBI. Aβ plaques are reported in up to 30% of post-mortem TBI patients. Aβ can accumulate rapidly following TBI, reported as early as 2–4 h (Graham et al., 1996). Plaque formation is described as diffuse plaques seen in early-stage AD and seen in all ages, even children, but determined to be more robust in elderly affected individuals (Johnson et al., 2012). Post-mortem TBI brains show greater Aβ density compared to age-matched controls years following injury (Johnson et al., 2012). Notably, Aβ42 is seen to be the major type following a TBI event in brain and CSF (Breunig et al., 2013). Interestingly, Aβ can accumulate in the white matter following TBI, unlike AD where it is more prominent in gray matter. It is described that the key constituents that generate Aβ (APP, β-secretase, and γ-secretase) anomalously colocalize at swollen and disconnected axonal bulb sites, producing and releasing Aβ into the brain parenchyma (Chen et al., 2009). Repetitive mTBI is associated to other tauopathies, especially to chronic traumatic encephalopathy (CTE). NFT pathology was found in 8 out of 27 post-mortem TBI brains (Smith et al., 2003). In fact, widespread NFTs are present in up to a third of patients following survival of a year or more from a single TBI (Johnson et al., 2012). Tau levels in CSF from TBI patients expressed over a 1,000-fold increase compared to various neurological-diseased and non-diseased individuals (Zemlan et al., 1999). Alterations of multiple protein kinases and phosphatases, neurofilament proteins, APP, BACE1, and even aggregated α-synuclein have been also found after TBI (Uryu et al., 2007). Therefore, TBI induces disease processes that may accelerate the formation and aggregation of misfolded proteins, possibly through axonal damage, and perhaps building upon the pathogenesis of AD.

### Epilepsy

Epilepsy can be defined as a neurological disorder where there is a continual and spontaneous propensity to have seizure activity as convulsions or non-convulsions due to abnormal neural firing and networks. Genes, developmental mechanisms, injury insult, and neuronal plasticity are thought to play chief roles in epilepsy (Scharfman, 2007). The convoluted functional neuronal network imbalances in epilepsy can ultimately result in neuropathological changes, brain atrophy, and cognitive decline (Friedman et al., 2012). It is not known if epileptic seizures are a cause or an effect of AD, but certainly, they both share mutual molecular and cellular mechanisms (Bazil, 2003). MCI and early-stage AD patients with epileptic activity show earlier onset of AD and accelerated rate of cognitive decline (Vossel et al., 2013, 2016; Cretin et al., 2016). On the other hand, multiple studies indicate that AD patients have an elevated risk of developing seizures or epilepsy (Vossel et al., 2016). Indeed, the pervasiveness of seizures is about 7–8 fold higher in individuals with AD than individuals without dementia (Amatniek et al., 2006). Additional studies posit that the younger the age of dementia onset is correlated to increased seizure risk, as well as the severity or stage of AD, has a parallel relationship with seizure (Horvath et al., 2016). Seizures are commonly described in cases of familial AD and strongly related to Down syndrome cases. Thus, epileptic seizures could be an early event in AD progression or an integral part of AD severity. However, more studies are needed as a systemic database meta-analysis between dementia and epilepsy concluded significant gaps of knowledge in epidemiology between the two disorders with insufficient data to pool an overall incidence rate.

Central neural circuits maintain a mean firing rate related to constant, spontaneous neuronal activity, which is dependent on intrinsic circuit excitability, and their synaptic properties and functional connectome. Firing instability and limited synapse flexibility at early AD stages trigger a vicious cycle and dysregulation of an integrated homeostatic network (Frere and Slutsky, 2018). Firing rates also can be dependent on the balance of excitation and inhibition ratio, and its imbalance could play a role in AD and epilepsy. Hyperexcitability can be triggered by the mismanagement of glutamate levels and Ca^2+^ homeostasis in the brain. Glial cells could also play a role in hyperexcitability and seizures as induced seizures are shown to excite astrocytes directly by stimulating the release of glial glutamate (Ding et al., 2007). Phase-coupling of oscillations in the brain is central for normal brain function. Gamma oscillations (30–150 Hz) are known to increase locally for sensory processing and memory encoding, while other oscillations would be reduced accordingly, such as alpha, beta, and theta. AD patients exhibit reductions in gamma power oscillations. Pharmacological inhibition of gamma oscillations leads to augmented epileptic activity in experimental animals (Maheshwari et al., 2016). Gamma oscillations can be increased by social interactions and mental stimulation; therefore, these activities have been suggested as a preventative measure in AD development. Antiepileptic treatments, such as levetiracetam, reverse neural network impairments and behavior (Sanchez et al., 2012), decrease brain dysrhythmia (Das et al., 2018), and improves cognition in animal models and MCI patients (Bakker et al., 2012).

A plethora of AD transgenic animal models reveals stochastic epileptiform. In general, Aβ is thought to induce neuronal hyperexcitability by differentially attacking excitatory and inhibitory neurons. Aβ can affect nACh, N-Methyl-D-aspartate (NMDA), and AMPA receptors, and calbindin pathways (Corbett et al., 2017). In APP23xPS45 mice, neuronal hyperexcitability occurs before any Aβ plaque deposition. Inhibition of oligomeric Aβ restores neural activity while inoculation of oligomeric Aβ in wild-type mice induces hyperexcitability (Busche et al., 2008). AD Tg mice also reveal cortical hyperactivity near amyloid plaques due to decreased GABAergic inhibition. APP/PS1 mice have early-impaired GABAergic interneurons in the hippocampus and entorhinal cortex (Ramos et al., 2006; Moreno-Gonzalez et al., 2009; Baglietto-Vargas et al., 2010). Administration of Aβ suppresses gamma oscillations *in vivo* and *in vitro* (Mucke and Selkoe, 2012). The chronic presence of Aβ and hyperexcitability effect could have an indirect effect by exhausting inhibitory neurons resulting in their deterioration. On the contrary, vulnerable neural networks produced by epileptogenic episodes could aid in the triggering of Aβ plaques. Chronic neural stimulation promotes amyloid deposition and elicits epileptic activity in an AD mouse model (Yamamoto et al., 2015), as well as increasing firing rate has been noted to surge Aβ production (Kamenetz et al., 2003). Tau protein could play a much larger role in neuronal activity and, therefore, epileptiform activity than previously thought. Epileptic patients present elevated levels of total tau in CSF (Monti et al., 2015). In fact, pharmacologically induced epilepsy in 3xTg-AD mice leads to elevated hyperphosphorylated tau levels in the dentate granule cells and mossy fibers (Yan et al., 2012), indicating the effect of epileptic activity in tau misfolding and aggregation. Moreover, synaptic activity can stimulate the release of tau and spreading of tau pathology, induce tau phosphorylation, and relocate tau to the dendritic spines (Khan et al., 2014; Frandemiche et al., 2014; Wu et al., 2016). On the other hand, reduction of tau protein levels prevents cognitive decline, synaptic impairment, and spontaneous epileptiform activity in several APP mouse models (Roberson et al., 2011; de Calignon et al., 2012). A152T tau transgenic mice present abnormal brain oscillations (Das et al., 2018), suggesting a mutual effect of epileptic activity and tau aggregation. Nevertheless, future studies are needed to elucidate epileptiform activity in the etiology and progression of AD; however, current research dictates a strong relationship between epilepsy and AD.

### Depression

Depression, also termed major depressive disorder, is a serious medical illness with a wide range of mental health issues that affects about 300 million people worldwide (World Health Organization, 2017). This disease is characterized by feelings of sadness and loss of interest in ordinary things. A common triad of symptoms seen include anhedonia, low energy or fatigue, and a low or depressed mood. Depression is a common symptom seen in people suffering from AD (Drevets and Rubin, 1989; Lyketsos et al., 1996). There is a debate whether depression is a risk factor for developing AD, rather than just a symptom. Recently, several clinical studies bolstered the idea of depressive symptoms as a crucial risk factor for cognitive decline and AD. It has been shown that the age of onset for AD is expedited in MCI patients with a history of depression (de Oliveira et al., 2015). In fact, there is a strong association between depression and AD onset (Barnes et al., 2012; Steenland et al., 2012). In addition, a less studied area between depression and AD is whether the age of onset of depression could lead to a different pathology of AD. Early-life depression (ELD) is characterized as onset before the age of 60 as opposite to late-life depression (LLD), so there is interest to determine how the age of onset of depression would influence the progression of AD. It remains to be elucidated if depression onset, whether ELD or LLD, could influence the progression of AD or even engender disparate pathology in AD. Large-scale prospective studies proposed that LLD, but not early or mid-life, increases the risk of AD (Barnes et al., 2012). These findings were confirmed by a recent meta-analysis study suggesting LLD increases the risk of AD incidence by 1.65-folds (Diniz et al., 2013). Moreover, a recent study reported elevated Aβ deposition in patients with a lifetime history of major depression (Li P. et al., 2017). Additionally, individuals with MCI plus coexistent depressive symptoms have an elevated Aβ load and a higher risk of faster conversion to AD compared to non-depressed MCI patients (Hebert et al., 2013).

Neurotransmitters like dopamine and serotonin play a crucial role in both the development of depression and in AD pathology (Chen et al., 1996; Jacobsen et al., 2012). Serotonin helps regulate mood, social behavior, and memory, whereas, dopamine, functions in motor control and reward-motivated behavior. Therefore, these two neurotransmitters could be key in the conversion of depression into AD. In AD mice, dopaminergic neuronal loss in the midbrain leads to memory impairment (Nobili et al., 2017), whereas restoration of dopamine release improves cognitive dysfunction (Guzman-Ramos et al., 2012). In depression, there is a decrease in dopamine production leading to a loss of reward-motivated and low levels of serotonin have been associated with depressive behavior since serotonin regulates mood and social behavior. Additionally, selective serotonin reuptake inhibitors or SSRIs reduce brain Aβ levels by increasing serotonin levels in the brain (Nelson et al., 2007).

Many factors can lead to the onset of depression, but the most studied cause is stress (Monroe et al., 2007; Ross et al., 2018). Stress works by activating the hypothalamus-pituitary-adrenal (HPA) axis leading to the release of glucocorticoid hormones from the adrenal cortex—cortisol in humans and corticosterone in rodents (Caruso et al., 2018). Rising levels of cortisol can negatively affect the HPA axis and refrain it from maintaining its sensitivity and regulating the stress response. Increased cortisol levels have been seen in biological fluids of patients affected by AD (Hatzinger et al., 1995; Rasmuson et al., 2001; Curto et al., 2017). Stress increases the production of Aβ and enhances the formation of amyloid plaques by increasing corticotropin-releasing factor release, which leads to an increase in neuronal activity, stimulating the production of Aβ, and demonstrating a link between depression and AD development (Dong and Csernansky, 2009). In addition, stress induces neuronal loss in the hippocampus, an area known to be one of the earliest regions affected by AD neuropathology, by increasing glucocorticoid release and decreasing neurotrophic factors (Kumamaru et al., 2008). Oral administration of corticosterone leads to morphological changes in the hippocampal region of rats, adding to the idea that stress triggers directed neurodegeneration (Magariños et al., 1998). Recently, patients suffering from LLD presented a faster hippocampal atrophy rate than those with ELD, eluding that LLD, and not ELD, leads to a higher risk of developing AD. Therefore, there may be a different progression of the disease depending on the age of depression onset (Taylor et al., 2014), and LLD could be considered a risk factor for AD development. The effect of taking antidepressants on AD pathology and development before the onset of the disease as a preventive therapy in LLD population remains to be determined.

## Lifestyle

### Physical Activity

Under normal circumstances, an elderly individual without dementia diagnosis will exhibit hippocampal volume shrinkage of 1%–2% each year (Erickson et al., 2011). Hippocampal shrinkage may be reversed by a moderate-intensity exercise training. A 1-year aerobic exercise intervention was effective at increasing hippocampal volume by 2% and offsetting normal decline associated with aging (Erickson et al., 2011) and individuals with life-long exercise routine reveal larger brain volume and improved executive function than inactive older adults (Tseng et al., 2013). However, the increase in volume was selective since it only influenced the anterior hippocampus including the dentate gyrus, in which cell proliferation occurs. Clinical studies indicate that physical activity may be neuroprotective by preserving cognition and maintaining the brain neuroplasticity (Kramer et al., 1999; Winter et al., 2007). In addition to prevention, exercise has shown to have a favorable outcome on improving cognitive symptoms. AD patients performing a moderate exercise program for a year exhibited a slower decline in the capability to achieve activities of daily living and amelioration on the physical impairment (Rolland et al., 2007; Pitkälä et al., 2013). Some other studies have also found that aerobic exercise is able to improve memory performance and cognitive function in aging, MCI, and AD patients (Baker et al., 2010; Vidoni et al., 2012; Morris et al., 2017). Although all these studies indicate that exercise may be effective in reducing the clinical symptoms observed in AD patients, there are no studies reporting its effect on amyloid deposition and how physical activity may prevent from developing AD in at risk population. In this direction, several studies have intended to investigate the beneficial effects of exercise on cognitive function and amyloid deposition in AD models. Streptozotocin-induced mice where placed on treadmill exercise daily for 30 min for a month. Afterwards, rats showed a decline in amyloidogenesis and tauopathy as well as a suppression of neuroinflammation and oxidative stress leading to selective anti-inflammatory microglia activation and pro-inflammatory microglia inhibition, hippocampal neuroprotection, and overall cognitive preservation (Lu et al., 2017). In transgenic animal models of AD, exercise leads to amelioration of behavior impairment, reduction of Aβ deposition, lager hippocampal volume and decreased apoptosis (Adlard et al., 2005; Nichol et al., 2007; Um et al., 2008; Liu et al., 2013), especially in animals under a voluntary exercise routine (Yuede et al., 2009). Enhanced cognitive function was also observed in ApoEε4 mouse models (Nichol et al., 2009). Likewise, Tau transgenic animals subjected to forced or voluntary treadmill exercise for several months show reduced levels of total tau, ptau and insoluble tau, although it had no neuroprotective effects (Leem et al., 2009; Belarbi et al., 2011; Ohia-Nwoko et al., 2014). Likewise, exercise decreases BACE (secretase beta-site APP cleaving enzyme-1) activity and APP levels compared to sedentary rats (Alkadhi and Dao, 2018). Regarding neuroprotection, both treadmill and swimming exercise are able to decrease caspase-3 expression and revers the Bax to Bcl-2 ratio observed in AD (Jin et al., 2014; Baek and Kim, 2016). Treadmill exercise also increases sirtuin-1 (SIRT-1), a modulator of neuronal survival, in a transgenic AD mouse (Koo et al., 2017) and enhances spatial memory in AD mice through upregulation of c-Fos, an indicator of neuronal activity expressed after depolarization (Jee et al., 2008).

Brain-derived neurotrophic factor (BDNF) serves as a mediator in neurogenesis as well as dendritic expansion, playing a vital role in memory formation. Acute exercise increases BDNF production in the brain (Neeper et al., 1995; Ferris et al., 2007). Modifications in BDNF serum correlate with changes in the hippocampal volume in MCI and Borba et al. (2016). AD models exposed to treadmill exercise show promotion of cell proliferation and amelioration of memory impairment observed by an increase in BDNF and TrkB levels (Liu et al., 2011; Kim et al., 2014; Sim, 2014). BDNF also enhances non-amyloidogenic APP processing by activating α-secretase and, therefore, reducing the amount of toxic Aβ peptides after voluntary exercise (Nigam et al., 2017). A recent report suggests that additional stimulation of adult hippocampal neurogenesis and increase in BDNF levels is necessary to induce the cognitive beneficial effect of exercise (Choi et al., 2018). Irisin is a myokine that is also released by physical exercise (Wrann et al., 2013). FNDC5/irisin prevents the binding of Aβ oligomers to neurons reducing its toxicity *in vitro* whereas irisin knockout mice present a deterioration in long-term potentiation and memory. In AD brains, irisin levels are reduced positing this myokine as a mediator of the beneficial effects of exercise in preventing or reducing the deleterious effects of AD pathology (Lourenco et al., 2019). Therefore, recent reports suggest that physical activity has a positive effect on synaptic plasticity, hippocampal shrinkage, and memory formation in animal models and, moreover, it can decrease the load of amyloid aggregates. Studies performed in AD patients indicate that exercise ameliorate some of the AD-related clinical symptoms and helps to decrease the progression of the disease. It still remains unknown whether exercise could diminish the risk to develop AD although studies performed in MCI patients may shed light to its potential benefits for prevention.

### Sleep Disturbance

The sleep-wake cycle refers to a 24-h daily sleep pattern, typically consisting of 16 h of being awake and 8 h of sleep. This cycle, controlled by the body’s circadian rhythm and sleep homeostasis, is important to many brain functions and plays a role in removing toxins from the brain that have accumulated throughout the day. A sleep cycle consists of stages N1, N2, and N3 non-rapid eye movement (NREM) sleep followed by REM sleep. During REM sleep, the brain is highly active as it is being rewired and is considered the most important part of the sleep-wake cycle. With aging, the sleep pattern is altered by a reduction in sleeping time and REM sleep. Sleep-wake cycle disturbances, including increased daytime sleep, reduced nocturnal sleep, and sleep fragmentation, are a common feature seen in AD patients (Bonanni et al., 2005; Moran et al., 2005). It is well established that in AD patients electroencephalograms are characteristic of increases in N1 and N2 NREM sleep and REM latency, and decreases in REM sleep, leading to an overall decrease in sleep duration (Loewenstein et al., 1982). Recent studies indicate that prolonged sleep duration could be indicative of at risk population (Westwood et al., 2017) and, in fact, NREM characteristics may provide evidence of an already deteriorated cognitive condition (Taillard et al., 2019). Due to the association between aging, cognition and sleep disorders, it has been proposed that sleep disturbances may lead to an increased risk for AD development (Roh et al., 2012). In fact, disorders in the sleep pattern have been related to an increased risk to develop cognitive deficiency, including MCI and dementia (Diem et al., 2016). On the other hand, deposition of Aβ seems to deteriorate sleep efficiency (time in bed spent asleep), especially during the preclinical stage (Ju et al., 2013).

An increase in the amount of light throughout the sleep-wake cycle leads to an increase in insoluble tau and memory impairment since continuous light input suppresses the production of the hormone melatonin that regulates the sleep-wake cycle (Di Meco et al., 2014). It has been shown that sleep increases the rate of Aβ clearance in the brain through the glymphatic system (Xie et al., 2013). In fact, the interstitial concentration of Aβ was higher in awake humans when compared to sleeping ones, indicating that wakefulness is associated with an increased production of Aβ (Bateman et al., 2006). The glymphatic system is able to clear waste products through convective bulk flow of interstitial fluid (ISF) that is facilitated by astrocytic aquaporin 4 (AQP4) water channels. Moreover, removal of these AQP4 channels led to reduced clearance of Aβ by 65%, suggesting the importance of these channels in removing unwanted waste from the brain (Iliff et al., 2012). During sleep, there is an increase in the interstitial space, which leads to an augmentation in the exchange between CSF and ISF, facilitating Aβ clearance. In fact, just one night of acute sleep deprivation increases the levels of Aβ in the brain, independently of ApoE genotype, indicating the direct effect of sleep in AD pathology (Shokri-Kojori et al., 2018). Additionally, sleep also led to a decrease in ISF tau levels, shown by ~90% increase during wakefulness and ~100% increase during sleep deprivation (Holth et al., 2019). Moreover, these results were also seen in the CSF of patients who were sleep deprived, leading to a 50% increase in CSF tau levels. Changes in sleep precede the onset of cognitive symptoms seen in AD patients, and sleep-wake cycle disturbances are proposed as one of the earliest symptoms seen in AD. Thus, disruptions in the sleep-wake cycle could be considered a risk factor for AD and early management of sleep-wake cycle disturbances could prevent or slow the subsequent pathology and later onset of AD. Several strategies can be considered to modulate the effect of sleep disturbances in dementia risk, from sleep drugs to physical exercise (McCleery et al., 2016; Law et al., 2019). Orexin is a neuropeptide that regulates wakefulness and is implicated in various sleep disorders, such as narcolepsy and cataplexy. Treatment to effectively block orexin receptors mitigated brain ISF levels of Aβ that were elevated due to wakefulness or sleep deprivation in AD mice (Kang et al., 2009; Roh et al., 2015).

### Diet

Compiling evidence suggests that a Mediterranean diet (MeDi) or Mediterranean-Dietary Approaches to Stop Hypertension diet (MIND) reduce the risk of developing MCI or AD (Morris et al., 2015). MeDi is highly popular on its preventive effects on AD (Scarmeas et al., 2007). A MeDi consists of a low intake of saturated fatty acids, such as meat and poultry; a low-to-moderate consumption of dairy products, such as cheese and yogurt; a moderate amount of alcohol, such as wine; and a high intake of vegetables, legumes, fruits, cereals, fish and unsaturated fatty acids. Studies performed in Spain, France, North America, and recently in Australia, demonstrated that the higher the adherence to a MeDi lowered the risk to contracting diseases associated as risk factors for AD (Tangney et al., 2011; Gardener et al., 2012) and protects against cognitive decline in elderly population, specifically episodic memory and global cognition (Trichopoulou et al., 2015; Valls-Pedret et al., 2015; Loughrey et al., 2017), indicating that MeDi may reduce Alzheimer’s risk. A 3-year brain imaging study evaluated the effects of a low to a high adherence MeDi on AD biomarkers in 30–60-year-old cognitive normal participants. The study concluded that a higher MeDi adherence provided an average of 1.5–3.5 years of protection against AD as well as a lower adherence highlighted important AD biomarkers (Berti et al., 2018). MeDi has been shown to reduce oxidative stress by decreasing intracellular reactive oxidative species, apoptosis, and cells containing telomere shortening. Elderly patients adhering highly to MeDi demonstrated longer telomere length and high telomerase activity (Boccardi et al., 2013), and high intake of vegetables is also directly associated with longer telomere length (Gu et al., 2015). Several studies point out that the polyphenols found in the characteristic olive oil in the MeDi regimen are the main active components to prevent AD (Omar et al., 2018). Oleuropein aglycone, present in extra virgin olive oil, induces autophagy, decreases the amount of aggregated proteins, decreases inflammation, and improves cognitive function seen in AD (Grossi et al., 2013; Cordero et al., 2018). Hydrocytyrosol, another olive oil product, has antioxidant and anti-inflammatory properties. In APP/PS1 mice, hydrocytyrosol administration reduces mitochondrial oxidative stress, neuronal inflammation and apoptosis (Peng et al., 2016).

On the contrary, a high-fat diet (HFD) raises the risk of developing obesity, leading to increased chances to develop diabetes and, therefore, promoting the development of cognitive deficits, and perhaps AD. A HFD consists of a regimen where the majority of the calories ingested come from a fat source rather than carbohydrates or protein. AD transgenic mice fed using HFD for 4 months presented significant memory impairment compared with AD mice eating regular diet (Sah et al., 2017). There were no differences in the levels of Aβ or ptau suggesting that HFD induces cognitive impairments in an amyloid-independent pathway. In fact, HFD has shown to accelerate age-associated cognitive decline by decreasing BDNF levels, inducing oxidative stress, and generating a loss in synaptic plasticity (Thériault et al., 2016).

As already mentioned, insulin resistance, impaired glucose metabolism, and T2D are well known risk factors for AD. These conditions can be developed following a diet of high sugars, carbohydrates and glycemic loads. High-glycemic diet includes: high-sugar beverages and foods, white pasta and rice, French fries and baked potatoes, cereals with added sugar, and sundried fruit. A high-glycemic regimen correlates with an increment in Aβ accumulation before AD manifestation (Taylor et al., 2017). Consumption of fish oil and an omega-3 fatty acid-rich diet decreases plasma arachidonic acid/docosahexaenoic acid (AA/DHA) ratio levels. MCI and AD patients carrying ApoEε4 present increased AA/DHA ratio compared to carriers who did not present any cognitive deficiencies (Abdullah et al., 2017). DHA demonstrates a pleiotropic effect by balancing cell signal pathways, synaptic plasticity, and the enzymatic processing of Aβ (Davinelli et al., 2012). Fish oil/omega-3 fatty acid was correlated with a decline in AA/DHA levels and even in AD-ApoEε4 patients (Abdullah et al., 2017), indicating that omega-3 supplementation could be considered as an intervention against the risk of acquired AD, especially in ApoEε4 carriers. Whole food diet (WFD) consists of freeze-dried fish, fruits, and vegetables. AD mice fed with the WFD were highly impaired in spatial memory compared to controls and produced an elevated neuroinflammatory response (Parrott et al., 2015).

A large body of literature suggests that a balanced diet, full of fruits, vegetables, and lean meat and fish along with low sugar and high good fat content may be beneficial against cognitive impairment and, therefore, decrease the chances to develop AD. However, recent studies have found no significant association between dietary patterns, including MeDi, and risk for dementia (Haring et al., 2016; Akbaraly et al., 2019), indicating that adherence to healthy dietary patterns may not be enough to reduce the risk to develop age-related cognitive impairment and dementia.

### Smoking

Worldwide, approximately 1 billion people use tobacco products in the form of cigarettes, and annually, there are at least 6 million global deaths caused by tobacco-smoking related diseases (World Health Organization, 2013). Today, smoking-related incidence has expanded from including CVD and stroke to now including neurocognitive abnormalities (Swan and Lessov-Schlaggar, 2007; Durazzo et al., 2010). Smoking leads to cognitive impairment and decline shown by faster declines in verbal memory and slower visual search speeds (Richards et al., 2003). Additionally, cognitive decline in smokers is directly proportional to the number of packs they smoke per day (Kalmijn et al., 2002). Indeed, it is known that smoking has negative effects on cardiovascular diseases which, as mentioned, are risk factors of AD, stressing out the deleterious importance of smoking in promoting dementia.

Historically, smoking has been considered a preventative measure from developing AD as many have stated that nicotine improves short-term cognitive performances and inhibits amyloid formation (Brenner et al., 1993; Lee, 1994). Actually, nicotine has been proven to reduce APP secretion (Lahiri et al., 2002), inhibit Aβ aggregation (Dickerson and Janda, 2002), and reduce Aβ load in AD transgenic mice independently from inflammation (Nordberg et al., 2002; Hellström-Lindahl et al., 2004). However, more recent studies have questioned this evidence and indicate that smoking increases the chance to develop dementia and cognitive decline (Ott et al., 1998; Anstey et al., 2007; Reitz et al., 2007). Confirming this negative effect, nicotine exacerbates tau phosphorylation in experimental animals (Oddo et al., 2005). In addition, exposure to cigarette smoke exacerbates Alzheimer’s-like pathology by increasing amyloid deposition, inducing tau hyperphosphorylation, and exacerbating the inflammatory response in a smoke-consumption concentration dependent manner (Moreno-Gonzalez et al., 2013). Still some studies indicate that when epidemiological data is adjusted for competing risk of death without dementia, smoking seems not to be associated with dementia development (Abner et al., 2018), and, surprisingly, ApoEε4 carrier smokers are at a lower risk of developing dementia than smokers without this allele (Reitz et al., 2007). The mechanism by which smoking may lead to an increased risk in AD development is uncertain, and further studies need to be conducted for potential mechanisms responsible for a possible increased risk of AD development.

### Alcohol

Alcohol consumption is considered a major risk factor for many health problems. Heavy drinking is defined as: consuming more than four drinks a day (or 14 drinks a week) for males and consuming more than three drinks a day (or seven drinks a week) for females (Rehm, 2011). Knowing that alcohol negatively affects cognitive and motor functions, there is no surprise that heavy drinking has been associated with an increased risk of AD, whereas mild to moderate alcohol intake has been associated with a lower risk (Heymann et al., 2016). The extent to which alcohol affects AD pathology is still debated today as some believe that alcohol is protective of AD development (Luchsinger et al., 2004), while others believe the contrary (Piazza-Gardner et al., 2013), the latter being the strongest current of opinion. Heavy alcohol consumption leads to a decline in cognitive performance similar to that observed in AD (Weissenborn and Duka, 2003). Loss of cholinergic neurons observed in AD patients has also been reported in individuals exposed to ethanol consumption (Fernandez and Savage, 2017; Vetreno and Crews, 2018) as well as hippocampal atrophy (Topiwala et al., 2017), linking heavy alcohol consumption with cognitive impairment that may eventually trigger AD development. In fact, a combination of both smoking and drinking can have a more impactful effect of AD incidence than just one of those habits (Zhou et al., 2014). Some recent reports indicate that alcohol use is not associated with prodromal AD or disease progression (Heffernan et al., 2016; Bos et al., 2017). In addition to the potential link to trigger dementia, alcohol abstinence after AD diagnosis seems to ameliorate the cognitive damage initially observed (Toda et al., 2013), suggesting the alcohol consumption can, not just increase the risk of AD, but also worsen the progression of the disease in heavy intake conditions (Heymann et al., 2016). A potential mechanism proposed for alcohol to induce AD is by decreasing glymphathic function (Lundgaard et al., 2018). The glymphatic system plays an important part in removing brain waste, including Aβ. Since alcohol decreases glymphatic function, heavy drinking could induce Aβ accumulation by reducing its clearance triggering the cognitive abnormalities that are seen in alcohol use and AD.

On the other hand, alcoholic beverages such as wine—particularly red—contain polyphenols including morin, quercetin, resveratrol, and tannins, that are able to inhibit amyloid aggregation and can have other beneficial effects including reduction of oxidative stress, inflammation, and balance of protein homeostasis (Dhouafli et al., 2018). In fact, moderate drinking (1–2 drinks/day) has been proposed to be protective against AD by reducing amyloid burden, decreasing mortality, and reducing the risk of dementia (Russo et al., 2003; Deng et al., 2006; Wang et al., 2006), being low doses of wine the most recommended to reduce the risk of dementia (Xu et al., 2017). In AD animal models, low doses of ethanol decrease Aβ-mediated synaptic toxicity by direct interaction with Aβ peptide (Muñoz et al., 2015). This may be an indication that the amount (dinks/day), length of consumption, period of consumption (early or late life), and type of alcoholic beverages (fermented or distilled drinks) should be taken into consideration to determine the effect of alcohol consumption to protect or induce AD dementia.

## Concluding Remarks

Despite over a century since discovering AD, there is no cure that can halt, slow down, or reverse the progression of this neurodegenerative disease. Regardless of the tremendous effort that the scientific field has done to find effective treatments for AD, promising candidates fail when tested in AD patients (Cummings et al., 2007; Raschetti et al., 2007; Extance, 2010; van Dyck, 2018). Most of the recent clinical trials that attempt treating AD focus on inhibiting the main known culprits of AD. The central targets are amyloid production and aggregation, largely Aβ by immune therapy and pharmacological enzyme inhibition (BACE inhibitors); the use of NSAIDs to reduce inflammation; and even stem cell therapy to fight against neurodegeneration. However, all these attempts have failed probably because the therapeutic intervention was done in an already very advanced pathology or because the treatment is directed to the wrong target (Mehta et al., 2017). It could also well be that the proposed approach is targeting only one of the players of this multifactorial disease. Therefore, an alternative strategy to fight against AD could be the prevention of the known modifiable risk factors and related mechanisms for the disease. This includes proper management of comorbidities associated such as vascular diseases (hypertension, CVD, stroke, ischemia), diabetes, epilepsy, brain injuries, and depression as well as modification of lifestyle and avoidance of deleterious habits. Here, we have reviewed many of them including: physical activity, sleep, diet, and use of tobacco and alcohol, and how by different mechanisms, these factors are able to reduce amyloid deposition and ameliorate cognitive impairment. Recent studies have estimated that intervention of several modifiable risk factors could prevent up to 35% of dementia cases (Livingston et al., 2017). Management of diet, exercise, and vascular risk in at-risk elderly population can, in fact, prevent cognitive functioning deterioration (Kivipelto et al., 2013; Ngandu et al., 2015; Soininen et al., 2017). However, some reports suggest that changes in these habits should be done early in life since modifying later life lifestyle factors may not decrease the conversion of MCI to AD dementia (Reijs et al., 2017) although it may ameliorate the course of the disease. Most of the studies compiled in this review evaluate the effect of risk factors when the AD-associated pathological changes are already present, but very few analyze the potential of those factors in preventing the onset of the disease, rather than the further development. Furthermore, most of the risk factors analyzed here can be considered both the cause and effect of AD. If they are a cause or risk factor, preclinical intervention may prevent the onset and development of AD, but if these factors are in fact an effect or a symptom, their treatment will still slow down the progression of the disease. Hence intervention of potential risks is highly recommended for either prevention or even amelioration of clinical symptoms of AD since most of these actions will also benefit general health status. Therefore, and while an effective treatment(s) is developed to treat AD, the most reasonable approach is to prevent AD onset by managing multiple risk factors way before any clinical symptom is observed.

## Author Contributions

GEd, NG, GEs and OC drafted the article. IM-G provided a critical revision and generated the last version of the manuscript.

## Conflict of Interest Statement

The authors declare that the research was conducted in the absence of any commercial or financial relationships that could be construed as a potential conflict of interest.

## References

[B1] Abdullah L. Evans J. E. Emmerich T. Crynen G. Shackleton B. Keegan A. P.. (2017). APOE ε4 specific imbalance of arachidonic acid and docosahexaenoic acid in serum phospholipids identifies individuals with preclinical mild cognitive impairment/Alzheimer’s disease. Aging 9, 964–985. 10.18632/aging.101203 28333036 PMC5391242

[B2] Abedini A. Schmidt A. M. States U. (2015). Mechanisms of islet amyloidosis toxicity in type 2 diabetes. FEBS Lett. 587, 1119–1127. 10.1016/j.febslet.2013.01.017 23337872 PMC4557799

[B109] Abner E. L. Nelson P. T. Jicha G. A. Fardo D. W. Schmitt F. A. Kryscio R. J. (2018). Cigarette smoking and risk of dementia in a Kentucky cohort: a competing risk analysis. Alzheimers Dement. 14:973 10.1016/j.jalz.2018.06.1306 PMC6915304 30856115

[B3] Adlard P. A. Perreau V. M. Pop V. Cotman C. W. (2005). Voluntary exercise decreases amyloid load in a transgenic model of Alzheimer’s disease. J. Neurosci. 25, 4217–4221. 10.1523/JNEUROSCI.0496-05.2005 15858047 PMC6725122

[B4] Air E. L. Kissela B. M. (2007). Diabetes, the metabolic syndrome, and ischemic stroke: epidemiology and possible mechanisms. Diabetes Care 30, 3131–3140. 10.2337/dc06-1537 17848611

[B5] Akbaraly T. N. Singh-Manoux A. Dugravot A. Brunner E. J. Kivimäki M. Sabia S. (2019). Association of midlife diet with subsequent risk for dementia. J. Am. Med. Assoc. 321, 957–968. 10.1001/jama.2019.1432 30860560 PMC6436698

[B6] Alkadhi K. A. Dao A. T. (2018). Exercise decreases BACE and APP levels in the hippocampus of a rat model of Alzheimer’s disease. Mol. Cell. Neurosci. 86, 25–29. 10.1016/j.mcn.2017.11.008 29128320

[B7] Alosco M. L. Brickman A. M. Spitznagel M. B. Garcia S. L. Narkhede A. Griffith E. Y.. (2013). Cerebral perfusion is associated with white matter hyperintensities in older adults with heart failure. Congest. Heart Fail. 19, E29–E34. 10.1111/chf.12025 23517434 PMC3692594

[B8] Amatniek J. C. Hauser W. A. Delcastillo-Castaneda C. Jacobs D. M. Marder K. Bell K.. (2006). Incidence and predictors of seizures in patients with Alzheimer’s disease. Epilepsia 47, 867–872. 10.1111/j.1528-1167.2006.00554.x 16686651

[B9] Anstey K. J. von Sanden C. Salim A. O’Kearney R. (2007). Smoking as a risk factor for dementia and cognitive decline: a meta-analysis of prospective studies. Am. J. Epidemiol. 166, 367–378. 10.1093/aje/kwm116 17573335

[B10] Arvanitakis Z. Leurgans S. E. Wang Z. Wilson R. S. Bennett D. A. Schneider J. A. (2011). Cerebral amyloid angiopathy pathology and cognitive domains in older persons. Ann. Neurol. 69, 320–327. 10.1002/ana.22112 21387377 PMC3228518

[B11] Baek S.-S. Kim S.-H. (2016). Treadmill exercise ameliorates symptoms of Alzheimer disease through suppressing microglial activation-induced apoptosis in rats. J. Exerc. Rehabil. 12, 526–534. 10.12965/jer.1632858.429 28119873 PMC5227313

[B12] Baglietto-Vargas D. Moreno-Gonzalez I. Sanchez-Varo R. Jimenez S. Trujillo-Estrada L. Sanchez-Mejias E.. (2010). Calretinin interneurons are early targets of extracellular amyloid-β pathology in PS1/AβPP Alzheimer mice hippocampus. J. Alzheimers Dis. 21, 119–132. 10.3233/jad-2010-100066 20413859

[B13] Baglietto-Vargas D. Shi J. Yaeger D. M. Ager R. LaFerla F. M. (2016). Diabetes and Alzheimer’s disease crosstalk. Neurosci. Biobehav. Rev. 64, 272–287. 10.1016/j.neubiorev.2016.03.005 26969101

[B14] Baker L. D. Frank L. L. Foster-Schubert K. Green P. S. Wilkinson C. W. McTiernan A.. (2010). Effects of aerobic exercise on mild cognitive impairment: a controlled trial. Arch. Neurol. 67, 71–79. 10.1001/archneurol.2009.307 20065132 PMC3056436

[B15] Bakker A. Krauss G. L. Albert M. S. Speck C. L. Jones L. R. Stark C. E.. (2012). Reduction of hippocampal hyperactivity improves cognition in amnestic mild cognitive impairment. Neuron 74, 467–474. 10.1016/j.neuron.2012.03.023 22578498 PMC3351697

[B16] Ballard C. Gauthier S. Corbett A. Brayne C. Aarsland D. Jones E.. (2011). Alzheimer’s disease. Lancet 377, 1019–1031. 10.1016/S0140-6736(10)61349-9 21371747

[B17] Barbero-Camps E. Roca-Agujetas V. Bartolessis I. de Dios C. Fernández-Checa J. C. Marí M.. (2018). Cholesterol impairs autophagy-mediated clearance of amyloid β while promoting its secretion. Autophagy 14, 1129–1154. 10.1080/15548627.2018.1438807 29862881 PMC6103708

[B18] Barnes D. E. Yaffe K. Byers A. L. McCormick M. Schaefer C. Whitmer R. A. (2012). Midlife vs late-life depressive symptoms and risk of dementia: differential effects for Alzheimer disease and vascular dementia. Arch. Gen. Psychiatry 69, 493–498. 10.1001/archgenpsychiatry.2011.1481 22566581 PMC3704214

[B19] Bateman R. J. Munsell L. Y. Morris J. C. Swarm R. Yarasheski K. E. Holtzman D. M. (2006). Human amyloid-β synthesis and clearance rates as measured in cerebrospinal fluid *in vivo*. Nat. Med. 12, 856–861. 10.1038/nm1438 16799555 PMC2983090

[B20] Baumgart M. Snyder H. M. Carrillo M. C. Fazio S. Kim H. Johns H. (2015). Summary of the evidence on modifiable risk factors for cognitive decline and dementia: a population-based perspective. Alzheimers Dement. 11, 718–726. 10.1016/j.jalz.2015.05.016 26045020

[B21] Bazil C. W. (2003). Epilepsy and sleep disturbance. Epilepsy Behav. 4, 39–45. 10.1016/j.yebeh.2003.07.005 14527482

[B22] Beach T. G. Wilson J. R. Sue L. I. Newell A. Poston M. Cisneros R.. (2007). Circle of Willis atherosclerosis: association with Alzheimer’s disease, neuritic plaques and neurofibrillary tangles. Acta Neuropathol. 113, 13–21. 10.1007/s00401-006-0136-y 17021755

[B23] Belarbi K. Burnouf S. Fernandez-Gomez F. J. Laurent C. Lestavel S. Figeac M.. (2011). Beneficial effects of exercise in a transgenic mouse model of Alzheimer’s disease-like Tau pathology. Neurobiol. Dis. 43, 486–494. 10.1016/j.nbd.2011.04.022 21569847

[B24] Bellou V. Belbasis L. Tzoulaki I. Middleton L. T. Ioannidis J. P. A. Evangelou E. (2017). Systematic evaluation of the associations between environmental risk factors and dementia: an umbrella review of systematic reviews and meta-analyses. Alzheimers Dement. 13, 406–418. 10.1016/j.jalz.2016.07.152 27599208

[B25] Benedictus M. R. van Harten A. C. Leeuwis A. E. Koene T. Scheltens P. Barkhof F.. (2015). White matter hyperintensities relate to clinical progression in subjective cognitive decline. Stroke 46, 2661–2664. 10.1161/strokeaha.115.009475 26173729

[B26] Berhanu W. M. Yaşar F. Hansmann U. H. E. (2013). *In silico* cross seeding of Aβ and amylin fibril-like oligomers. ACS Chem. Neurosci. 4, 1488–1500. 10.1021/cn400141x 24007594 PMC3837374

[B27] Berti V. Walters M. Sterling J. Quinn C. G. Logue M. Andrews R.. (2018). Mediterranean diet and 3-year Alzheimer brain biomarker changes in middle-aged adults. Neurology 90, e1789–e1798. 10.1212/wnl.0000000000005527 29653991 PMC5957301

[B28] Blennow K. Brody D. L. Kochanek P. M. Levin H. McKee A. Ribbers G. M.. (2016). Traumatic brain injuries. Nat. Rev. Dis. Primers 2:16084. 10.1038/nrdp.2016.84 27853132

[B30] Boccardi V. Esposito A. Rizzo M. R. Marfella R. Barbieri M. Paolisso G. (2013). Mediterranean diet, telomere maintenance and health status among elderly. PLoS One 8:e62781. 10.1371/journal.pone.0062781 23646142 PMC3640022

[B31] Bonanni E. Maestri M. Tognoni G. Fabbrini M. Nucciarone B. Manca M. L.. (2005). Daytime sleepiness in mild and moderate Alzheimer’s disease and its relationship with cognitive impairment. J. Sleep Res. 14, 311–317. 10.1016/j.sleep.2005.05.001 16120107

[B32] Borba E. M. Duarte J. A. Bristot G. Scotton E. Camozzato A. L. Chaves M. L. F. (2016). Brain-derived neurotrophic factor serum levels and hippocampal volume in mild cognitive impairment and dementia due to alzheimer disease. Dement. Geriatr. Cogn. Dis. Extra 9, 559–567. 10.1159/000450601 28101102 PMC5216193

[B33] Bos I. Vos S. J. Frölich L. Kornhuber J. Wiltfang J. Maier W.. (2017). The frequency and influence of dementia risk factors in prodromal Alzheimer’s disease. Neurobiol. Aging 56, 33–40. 10.1016/j.neurobiolaging.2017.03.034 28482212

[B34] Brenner D. E. Kukull W. A. van Belle G. Bowen J. D. McCormick W. C. Teri L.. (1993). Relationship between cigarette smoking and Alzheimer’s disease in a population-based case-control study. Neurology 43, 293–300. 10.1212/wnl.43.2.293 8437692

[B35] Brenowitz W. D. Hubbard R. A. Keene C. D. Hawes S. E. Longstreth W. T. Jr. Woltjer R. L.. (2017). Mixed neuropathologies and estimated rates of clinical progression in a large autopsy sample. Alzheimers Dement. 13, 654–662. 10.1016/j.jalz.2016.09.015 27870939 PMC5438283

[B29] Bretherton-Watt D. Bloom S. R. (1991). Islet amyloid polypeptide: the cause of type-2 diabetes? Trends Endocrinol. Metab. 2, 203–206. 10.1016/1043-2760(91)90025-I 18411183

[B36] Breunig J. J. Guillot-Sestier M. V. Town T. (2013). Brain injury, neuroinflammation and Alzheimer’s disease. Front. Aging Neurosci. 5:26. 10.3389/fnagi.2013.00026 23874297 PMC3708131

[B300] Busche M. A. Eichhoff G. Adelsberger H. Abramowski D. Wiederhold K.-H. Haass C.. (2008). Clusters of hyperactive neurons near amyloid plaques in a mouse model of Alzheimer’s Disease. Science 321, 1686–1689. 10.1126/science.1162844 18802001

[B37] Caruso A. Nicoletti F. Mango D. Saidi A. Orlando R. Scaccianoce S. (2018). Stress as risk factor for Alzheimer’s disease. Pharmacol. Res. 132, 130–134. 10.1016/j.phrs.2018.04.017 29689315

[B38] Centers for Disease Control and Prevention (2017). National Diabetes Statistics Report. Atlanta, GA: Centers for Disease Control and Prevention, U.S. Dept of Health and Human Services.

[B39] Chatterjee S. Peters S. A. E. Woodward M. Mejia Arango S. Batty G. D. Beckett N.. (2016). Type 2 diabetes as a risk factor for dementia in women compared with men: a pooled analysis of 2.3 million people comprising more than 100,000 cases of dementia. Diabetes Care 39, 300–307. 10.2337/dc15-1588 26681727 PMC4722942

[B40] Chen C. P. Alder J. T. Bowen D. M. Esiri M. M. McDonald B. Hope T.. (1996). Presynaptic serotonergic markers in community-acquired cases of Alzheimer’s disease: correlations with depression and neuroleptic medication. J. Neurochem. 66, 1592–1598. 10.1046/j.1471-4159.1996.66041592.x 8627315

[B41] Chen X. H. Johnson V. E. Uryu K. Trojanowski J. Q. Smith D. H. (2009). A lack of amyloid β plaques despite persistent accumulation of amyloid β in axons of long-term survivors of traumatic brain injury. Brain Pathol. 19, 214–223. 10.1111/j.1750-3639.2008.00176.x 18492093 PMC3014260

[B42] Choi S. H. Bylykbashi E. Chatila Z. K. Lee S. W. Pulli B. Clemenson G. D.. (2018). Combined adult neurogenesis and BDNF mimic exercise effects on cognition in an Alzheimer’s mouse model. Science 361:eaan8821. 10.1126/science.aan8821 30190379 PMC6149542

[B43] Clark A. de Koning E. J. P. Hattersley A. T. Hansen B. C. Yajnik C. S. Poulton J. (1995). Pancreatic pathology in non-insulin dependent diabetes (NIDDM). Diabetes Res. Clin. Pract. 28, S39–S47. 10.1016/0168-8227(95)01075-o 8529518

[B44] Corbett B. F. You J. C. Zhang X. Pyfer M. S. Tosi U. Iascone D. M.. (2017). ΔFosB regulates gene expression and cognitive dysfunction in a mouse model of Alzheimer’s disease. Cell Rep. 20, 344–355. 10.1016/j.celrep.2017.06.040 28700937 PMC5785235

[B45] Cordero J. G. García-Escudero R. Avila J. Gargini R. García-Escudero V. (2018). Benefit of oleuropein aglycone for Alzheimer’s disease by promoting autophagy. Oxid. Med. Cell. Longev. 2018:5010741. 10.1155/2018/5010741 29675133 PMC5838478

[B47] Cretin B. Sellal F. Philippi N. Bousiges O. Di Bitonto L. Martin-Hunyadi C.. (2016). Epileptic prodromal Alzheimer’s disease, a retrospective study of 13 new cases: expanding the spectrum of Alzheimer’s disease to an epileptic variant? J. Alzheimers Dis. Park. 52, 1125–1133. 10.3233/JAD-150096 27104892

[B48] Cummings J. L. Doody R. Clark C. (2007). Disease-modifying therapies for Alzheimer disease: challenges to early intervention. Neurology 69, 1622–1634. 10.1212/01.wnl.0000295996.54210.69 17938373

[B49] Curto M. Martocchia A. Ferracuti S. Comite F. Scaccianoce S. Girardi P.. (2017). Increased total urinary cortisol (tUC) and serum brain-derived neurotrophic factor (BDNF) ratio in Alzheimer disease (AD)-affected patients. Alzheimer Dis. Assoc. Disord. 31, 173–176. 10.1097/wad.0000000000000156 27196536

[B50] Das M. Maeda S. Hu B. Yu G. Q. Guo W. Lopez I.. (2018). Neuronal levels and sequence of tau modulate the power of brain rhythms. Neurobiol. Dis. 117, 181–188. 10.1016/j.nbd.2018.05.020 29859869

[B51] Davinelli S. Sapere N. Zella D. Bracale R. Intrieri M. Scapagnini G. (2012). Pleiotropic protective effects of phytochemicals in Alzheimer’s disease. Oxid. Med. Cell. Longev. 2012:386527. 10.1155/2012/386527 22690271 PMC3368517

[B52] de Calignon A. Polydoro M. Suárez-Calvet M. William C. Adamowicz D. H. Kopeikina K. J.. (2012). Propagation of tau pathology in a model of early Alzheimer’s disease. Neuron 73, 685–697. 10.1016/j.neuron.2011.11.033 22365544 PMC3292759

[B53] de la Torre J. (2018). The vascular hypothesis of Alzheimer’s disease: a key to preclinical prediction of dementia using neuroimaging. J. Alzheimers Dis. 63, 35–52. 10.3233/JAD-180004 29614675

[B54] de Oliveira F. Bertolucci P. F. Chen E. Smith M. (2015). Assessment of risk factors for earlier onset of sporadic Alzheimer’s disease dementia. Neurol. India 62, 625–630. 10.4103/0028-3886.149384 25591674

[B55] Deng J. Zhou D. H. D. Li J. Wang Y. J. Gao C. Chen M. (2006). A 2-year follow-up study of alcohol consumption and risk of dementia. Clin. Neurol. Neurosurg. 108, 378–383. 10.1016/j.clineuro.2005.06.005 16084641

[B56] Dhouafli Z. Cuanalo-Contreras K. Hayouni E. A. Mays C. E. Soto C. Moreno-Gonzalez I. (2018). Inhibition of protein misfolding and aggregation by natural phenolic compounds. Cell. Mol. Life Sci. 75, 3521–3538. 10.1007/s00018-018-2872-2 30030591 PMC11105286

[B57] Di Meco A. Joshi Y. B. Praticò D. (2014). Sleep deprivation impairs memory, tau metabolism and synaptic integrity of a mouse model of Alzheimer’s disease with plaques and tangles. Neurobiol. Aging 35, 1813–1820. 10.1016/j.neurobiolaging.2014.02.011 24629673

[B58] Dickerson T. J. Janda K. D. (2002). A previously undescribed chemical link between smoking and metabolic disease. Proc. Natl. Acad. Sci. U S A 99, 15084–15088. 10.1073/pnas.222561699 12403823 PMC137547

[B59] Dickstein D. L. Walsh J. Brautigam H. Stockton S. D. Jr. Gandy S. Hof P. R. (2010). Role of vascular risk factors and vascular dysfunction in Alzheimer’s disease. Mt. Sinai J. Med. 77, 82–102. 10.1002/msj.20155 20101718 PMC2918901

[B60] Diem S. J. Blackwell T. L. Stone K. L. Yaffe K. Tranah G. Cauley J. A.. (2016). Measures of sleep-wake patterns and risk of mild cognitive impairment or dementia in older women. Am. J. Geriatr. Psychiatry 24, 248–258. 10.1016/j.jagp.2015.12.002 26964485 PMC4807599

[B61] Ding S. Fellin T. Zhu Y. Lee S.-Y. Auberson Y. P. Meaney D. F.. (2007). Enhanced astrocytic Ca^2+^ signals contribute to neuronal excitotoxicity after status epilepticus. J. Neurosci. 27, 10674–10684. 10.1523/JNEUROSCI.2001-07.2007 17913901 PMC2917229

[B62] Diniz B. S. Butters M. A. Albert S. M. Dew M. A. Reynolds C. F. III. (2013). Late-life depression and risk of vascular dementia and Alzheimer’s disease: systematic review and meta-analysis of community-based cohort studies. Br. J. Psychiatry 202, 329–335. 10.1192/bjp.bp.112.118307 23637108 PMC3640214

[B63] Dong H. Csernansky J. G. (2009). Effects of stress and stress hormones on amyloid-β protein and plaque deposition. J. Alzheimers Dis. 18, 459–469. 10.3233/jad-2009-1152 19584430 PMC2905685

[B64] Drevets W. C. Rubin E. H. (1989). Psychotic symptoms and the longitudinal course of senile dementia of the Alzheimer type. Biol. Psychiatry 25, 39–48. 10.1016/0006-3223(89)90145-5 2912509

[B65] Durazzo T. C. Meyerhoff D. J. Nixon S. J. (2010). Chronic cigarette smoking: implications for neurocognition and brain neurobiology. Int. J. Environ. Res. Public Health 7, 3760–3791. 10.3390/ijerph7103760 21139859 PMC2996190

[B66] Edwards G. III. Moreno-Gonzalez I. Soto C. (2017). Amyloid-β and tau pathology following repetitive mild traumatic brain injury. Biochem. Biophys. Res. Commun. 483, 1137–1142. 10.1016/j.bbrc.2016.07.123 27492070

[B67] Ellis R. J. Caligiuri M. Galasko D. Thal L. J. (1996). Extrapyramidal motor signs in clinically diagnosed Alzheimer disease. Alzheimer Dis. Assoc. Disord. 10, 103–114. 10.1097/00002093-199601020-00008 8727172

[B68] Erickson K. I. Voss M. W. Prakash R. S. Basak C. Szabo A. Chaddock L.. (2011). Exercise training increases size of hippocampus and improves memory. Proc. Natl. Acad. Sci. U S A 108, 3017–3022. 10.1073/pnas.1015950108 21282661 PMC3041121

[B69] Extance A. (2010). Alzheimer’s failure raises questions about disease-modifying strategies. Nat. Rev. Drug Discov. 9, 749–751. 10.1038/nrd3288 20885394

[B206] Fann J. R. Ribe A. R. Pedersen H. S. Fenger-Grøn M. Christensen J. Benros M. E.. (2018). Long-term risk of dementia among people with traumatic brain injury in Denmark: a population-based observational cohort study. Lancet Psychiatry 5, 424–431. 10.1016/s2215-0366(18)30065-8 29653873

[B70] Fernandez G. M. Savage L. M. (2017). Adolescent binge ethanol exposure alters specific forebrain cholinergic cell populations and leads to selective functional deficits in the prefrontal cortex. Neuroscience 361, 129–143. 10.1016/j.neuroscience.2017.08.013 28807788 PMC5609857

[B71] Ferris L. T. Williams J. S. Shen C. L. (2007). The effect of acute exercise on serum brain-derived neurotrophic factor levels and cognitive function. Med. Sci. Sports Exerc. 39, 728–734. 10.1249/mss.0b013e31802f04c7 17414812

[B72] Frandemiche M. L. De Seranno S. Rush T. Borel E. Elie A. Arnal I.. (2014). Activity-dependent tau protein translocation to excitatory synapse is disrupted by exposure to amyloid-β oligomers. J. Neurosci. 34, 6084–6097. 10.1523/JNEUROSCI.4261-13.2014 24760868 PMC6608293

[B73] Frere S. Slutsky I. (2018). Alzheimer’s disease: from firing instability to homeostasis network collapse. Neuron 97, 32–58. 10.1016/j.neuron.2017.11.028 29301104

[B74] Friedman D. Honig L. S. Scarmeas N. (2012). Seizures and epilepsy in Alzheimer’s disease. CNS Neurosci. Ther. 18, 285–294. 10.1111/j.1755-5949.2011.00251.x 22070283 PMC3630499

[B75] Frölich L. Blum-Degen D. Bernstein H. G. Engelsberger S. Humrich J. Laufer S.. (1998). Brain insulin and insulin receptors in aging and sporadic Alzheimer’s disease. J. Neural Transm. 105, 423–438. 10.1007/s007020050068 9720972

[B76] Garcia-Alloza M. Gregory J. Kuchibhotla K. V. Fine S. Wei Y. Ayata C.. (2011). Cerebrovascular lesions induce transient-amyloid deposition. Brain 134, 3697–3707. 10.1093/brain/awr300 22120142 PMC3235567

[B77] Gardener S. Gu Y. Rainey-Smith S. R. Keogh J. B. Clifton P. M. Mathieson S. L.. (2012). Adherence to a Mediterranean diet and Alzheimer’s disease risk in an Australian population. Transl. Psychiatry 2:e164. 10.1038/tp.2012.91 23032941 PMC3565821

[B78] Geifman N. Brinton R. D. Kennedy R. E. Schneider L. S. Butte A. J. (2017). Evidence for benefit of statins to modify cognitive decline and risk in Alzheimer’s disease. Alzheimers Res. Ther. 9:10. 10.1186/s13195-017-0237-y 28212683 PMC5316146

[B79] Gentleman S. M. Leclercq P. D. Moyes L. Graham D. I. Smith C. Griffin W. S. T.. (2004). Long-term intracerebral inflammatory response after traumatic brain injury. Forensic Sci. Int. 146, 97–104. 10.1016/j.forsciint.2004.06.027 15542269

[B80] Gottesman R. F. Albert M. S. Alonso A. Coker L. H. Coresh J. Davis S. M.. (2017a). Associations between midlife vascular risk factors and 25-year incident dementia in the Atherosclerosis Risk in Communities (ARIC) cohort. JAMA Neurol. 74, 1246–1254. 10.1001/jamaneurol.2017.1658 28783817 PMC5710244

[B81] Gottesman R. F. Schneider A. L. C. Zhou Y. Coresh J. Green E. Gupta N.. (2017b). Association between midlife vascular risk factors and estimated brain amyloid deposition. JAMA 317, 1443–1450. 10.1001/jama.2017.3090 28399252 PMC5921896

[B82] Graham J. E. Rockwood K. Beattie B. L. McDowell I. Eastwood R. Gauthier S. (1996). Standardization of the diagnosis of dementia in the canadian study of health and aging. Neuroepidemiology 15, 246–256. 10.1159/000109914 8878077

[B83] Graves A. B. White E. Koepsell T. D. Reifler B. V. van Belle G. Larson E. B.. (1990). The association between head trauma and Alzheimer’s disease. Am. J. Epidemiol. 131, 491–501. 10.1093/oxfordjournals.aje.a115523 2405648

[B84] Grossi C. Rigacci S. Ambrosini S. Ed Dami T. Luccarini I. Traini C.. (2013). The polyphenol oleuropein aglycone protects TgCRND8 mice against Aß plaque pathology. PLoS One 8:e71702. 10.1371/journal.pone.0071702 23951225 PMC3738517

[B85] Gu Y. Honig L. S. Schupf N. Lee J. H. Luchsinger J. A. Stern Y.. (2015). Mediterranean diet and leukocyte telomere length in a multi-ethnic elderly population. Age 37:24. 10.1007/s11357-015-9758-0 25750063 PMC4352412

[B86] Guo J. Shou C. Meng L. Jiang B. Dong B. Yao L.. (2007). Neuronal protein synuclein γ predicts poor clinical outcome in breast cancer. Int. J. Cancer 121, 1296–1305. 10.1002/ijc.22763 17534899

[B87] Guzman-Ramos K. Moreno-Castilla P. Castro-Cruz M. McGaugh J. L. Martinez-Coria H. LaFerla F. M. (2012). Restoration of dopamine release deficits during object recognition memory acquisition attenuates cognitive impairment in a triple transgenic mice model of Alzheimer’s disease. Learn. Mem. 19, 453–460. 10.1101/lm.026070.112 22984283

[B88] Haglund M. Passant U. Sjöbeck M. Ghebremedhin E. Englund E. (2006). Cerebral amyloid angiopathy and cortical microinfarcts as putative substrates of vascular dementia. Int. J. Geriatr. Psychiatry 21, 681–687. 10.1002/gps.1550 16802283

[B89] Haring B. Wu C. Mossavar-Rahmani Y. Snetselaar L. Brunner R. Wallace R. B.. (2016). No association between dietary patterns and risk for cognitive decline in older women with 9-year follow-up: data from the women’s health initiative memory study. J. Acad. Nutr. Diet. 116, 921–930. 10.1016/j.jand.2015.12.017 27050728 PMC4884559

[B90] Hatzinger M. Z’Brun A. Hemmeter U. Seifritz E. Baumann F. Holsboer-Trachsler E.. (1995). Hypothalamic-pituitary-adrenal system function in patients with Alzheimer’s disease. Neurobiol. Aging 16, 205–209. 10.1016/0197-4580(94)00159-6 7777138

[B91] Hebert L. E. Weuve J. Scherr P. A. Evans D. A. (2013). Alzheimer disease in the United States (2010–2050) estimated using the 2010 census. Neurology 80, 1778–1783. 10.1212/WNL.0b013e31828726f5 23390181 PMC3719424

[B92] Heffernan M. Mather K. A. Xu J. Assareh A. A. Kochan N. A. Reppermund S.. (2016). Alcohol consumption and incident dementia: evidence from the sydney memory and ageing study. J. Alzheimers Dis. 52, 529–538. 10.3233/jad-150537 27031466

[B94] Hellström-Lindahl E. Court J. Keverne J. Svedberg M. Lee M. Marutle A.. (2004). Nicotine reduces Aβ in the brain and cerebral vessels of APPsw mice. Eur. J. Neurosci. 19, 2703–2710. 10.1111/j.0953-816X.2004.03377.x 15147304

[B95] Hemming M. L. Selkoe D. J. (2005). Amyloid β-protein is degraded by cellular angiotensin-converting enzyme (ACE) and elevated by an ACE inhibitor. J. Biol. Chem. 280, 37644–37650. 10.1074/jbc.M508460200 16154999 PMC2409196

[B96] Hemming M. L. Selkoe D. J. Farris W. (2007). Effects of prolonged angiotensin-converting enzyme inhibitor treatment on amyloid β-protein metabolism in mouse models of Alzheimer disease. Neurobiol. Dis. 26, 273–281. 10.1016/j.nbd.2007.01.004 17321748 PMC2377010

[B97] Heymann D. Stern Y. Cosentino S. Tatarina-Nulman O. Dorrejo J. N. Gu Y. (2016). The association between alcohol use and the progression of Alzheimer’s disease. Curr. Alzheimer Res. 13, 1356–1362. 10.2174/1567205013666160603005035 27628432 PMC5526221

[B98] Holth J. K. Fritschi S. K. Wang C. Pedersen N. P. Cirrito J. R. Mahan T. E.. (2019). The sleep-wake cycle regulates brain interstitial fluid tau in mice and CSF tau in humans. Science 363, 880–884. 10.1126/science.aav2546 30679382 PMC6410369

[B99] Hongpaisan J. Sun M.-K. Alkon D. L. (2011). PKC activation prevents synaptic loss, a elevation and cognitive deficits in Alzheimer’s disease transgenic mice. J. Neurosci. 31, 630–643. 10.1523/JNEUROSCI.5209-10.2011 21228172 PMC6623433

[B100] Horvath A. Szucs A. Barcs G. Noebels J. L. Kamondi A. (2016). Epileptic seizures in Alzheimer disease: a review. Alzheimer Dis. Assoc. Disord. 30, 186–192. 10.1097/wad.0000000000000134 26756385

[B101] Hu X. Song C. Fang M. Li C. (2018). Simvastatin inhibits the apoptosis of hippocampal cells in a mouse model of Alzheimer’s disease. Exp. Ther. Med 15, 1795–1802. 10.3892/etm.2017.5620 29434767 PMC5776644

[B102] Iadecola C. (2004). Neurovascular regulation in the normal brain and in Alzheimer’s disease. Nat. Rev. Neurosci. 5, 347–360. 10.1038/nrn1387 15100718

[B46] Iadecola C. (2013). The pathobiology of vascular dementia. Neuron 80, 844–866. 10.1016/j.neuron.2013.10.008 24267647 PMC3842016

[B103] Iliff J. J. Wang M. Liao Y. Plogg B. A. Peng W. Gundersen G. A.. (2012). A paravascular pathway facilitates CSF flow through the brain parenchyma and the clearance of interstitial solutes, including amyloid β. Sci. Transl. Med. 4:147ra111. 10.1126/scitranslmed.3003748 22896675 PMC3551275

[B104] Jacobsen J. Siesser W. B. Sachs B. D. Peterson S. Cools M. J. Setola V.. (2012). Deficient serotonin neurotransmission and depression-like serotonin biomarker alterations in tryptophan hydroxylase 2 (Tph2) loss-of-function mice. Mol. Psychiatry 17, 694–704. 10.1038/mp.2011.50 21537332 PMC3536482

[B105] Janelidze S. Stomrud E. Palmqvist S. Zetterberg H. van Westen D. Jeromin A.. (2016). Plasma β-amyloid in Alzheimer’s disease and vascular disease. Sci. Rep. 6:26801. 10.1038/srep26801 27241045 PMC4886210

[B106] Janson J. Laedtke T. Parisi J. E. O’Brien P. Petersen R. C. Butler P. C. (2004). Increased risk of type 2 diabetes in Alzheimer disease. Diabetes 53, 474–481. 10.2337/diabetes.53.2.474 14747300

[B107] Janson J. Soeller W. C. Roche P. C. Nelson R. T. Torchia A. J. Kreutter D. K.. (1996). Spontaneous diabetes mellitus in transgenic mice expressing human islet amyloid polypeptide. Proc. Natl. Acad. Sci. U S A 93, 7283–7288. 10.1073/pnas.93.14.7283 8692984 PMC38975

[B108] Jee Y.-S. Ko I.-G. Sung Y.-H. Lee J.-W. Kim Y.-S. Kim S.-E.. (2008). Effects of treadmill exercise on memory and c-Fos expression in the hippocampus of the rats with intracerebroventricular injection of streptozotocin. Neurosci. Lett. 443, 188–192. 10.1016/j.neulet.2008.07.078 18687381

[B110] Jin J.-J. Ko I.-G. Kim S.-E. Shin M.-S. Kim S.-H. Jee Y.-S. (2014). Swimming exercise ameliorates multiple sclerosis-induced impairment of short-term memory by suppressing apoptosis in the hippocampus of rats. J. Exerc. Rehabil. 10, 69–74. 10.12965/jer.140103 24877040 PMC4025552

[B111] Johnson V. E. Stewart W. Smith D. H. (2012). Widespread tau and amyloid-β pathology many years after a single traumatic brain injury in humans. Brain Pathol. 22, 142–149. 10.1111/j.1750-3639.2011.00513.x 21714827 PMC3979351

[B112] Ju Y. E. S. McLeland J. S. Toedebusch C. D. Xiong C. Fagan A. M. Duntley S. P.. (2013). Sleep quality and preclinical Alzheimer disease. JAMA Neurol. 70, 587–593. 10.1001/jamaneurol.2013.2334 23479184 PMC3676720

[B113] Jucker M. Walker L. C. (2015). Pathogenic protein seeding in Alzheimer’s disease and other neurodegenerative disorders. Annu. Rev. Neurosci. 38, 87–103. 10.1002/ana.22615 25840008 PMC4803040

[B114] Kalmijn S. van Boxtel M. P. J. Verschuren M. W. M. Jolles J. Launer L. J. (2002). Cigarette smoking and alcohol consumption in relation to cognitive performance in middle age. Am. J. Epidemiol. 156, 936–944. 10.1093/aje/kwf135 12419766

[B115] Kamenetz F. Tomita T. Hsieh H. Seabrook G. Borchelt D. Iwatsubo T. (2003). APP processing and synaptic function. Neuron 37, 925–937. 10.1016/S0896-6273(03)00124-7 12670422

[B116] Kang J. E. Lim M. M. Bateman R. J. Lee J. J. Smyth L. P. Cirrito J. R.. (2009). Amyloid-β dynamics are regulated by orexin and the sleep-wake cycle. Science 326, 1005–1007. 10.1126/science.1180962 19779148 PMC2789838

[B117] Kapurniotu A. Tatarek-Nossol M. Andreetto E. Frank R. Yan L.-M. Velkova A. (2010). Identification of hot regions of the Aβ-IAPP interaction interface as high-affinity binding sites in both cross- and self-association. Angew. Chem. Int. Ed Engl. 49, 3081–3085. 10.1002/anie.200904902 20309983

[B118] Khan U. A. Liu L. Provenzano F. A. Berman D. E. Profaci C. P. Sloan R.. (2014). Molecular drivers and cortical spread of lateral entorhinal cortex dysfunction in preclinical Alzheimer’s disease. Nat. Neurosci. 17, 304–311. 10.1038/nn.3606 24362760 PMC4044925

[B119] Kilander L. Andrén B. Nyman H. Lind L. Boberg M. Lithell H. (1998). Atrial fibrillation is an independent determinant of low cognitive function: a cross-sectional study in elderly men. Stroke 29, 1816–1820. 10.1161/01.str.29.9.1816 9731601

[B120] Kim B.-K. Shin M.-S. Kim C.-J. Baek S.-B. Ko Y.-C. Kim Y.-P. (2014). Treadmill exercise improves short-term memory by enhancing neurogenesis in amyloid β-induced Alzheimer disease rats. J. Exerc. Rehabil. 10, 2–8. 10.12965/jer.140086 24678498 PMC3952831

[B121] Kivipelto M. Solomon A. Ahtiluoto S. Ngandu T. Lehtisalo J. Antikainen R.. (2013). The finnish geriatric intervention study to prevent cognitive impairment and disability (FINGER): study design and progress. Alzheimers Dement. 9, 657–665. 10.1016/j.jalz.2012.09.012 23332672

[B122] Koo J.-H. Kang E.-B. Oh Y.-S. Yang D.-S. Cho J.-Y. (2017). Treadmill exercise decreases amyloid-β burden possibly via activation of SIRT-1 signaling in a mouse model of Alzheimer’s disease. Exp. Neurol. 288, 142–152. 10.1016/j.expneurol.2016.11.014 27889467

[B123] Kramer A. F. Hahn S. Cohen N. J. Banich M. T. McAuley E. Harrison C. R.. (1999). Ageing, fitness and neurocognitive function. Nature 400, 418–419. 10.1038/22682 10440369

[B124] Kumamaru E. Numakawa T. Adachi N. Yagasaki Y. Izumi A. Niyaz M.. (2008). Glucocorticoid prevents brain-derived neurotrophic factor-mediated maturation of synaptic function in developing hippocampal neurons through reduction in the activity of mitogen-activated protein kinase. Mol. Endocrinol. 22, 546–558. 10.1210/me.2007-0264 18096693 PMC5419617

[B125] Kwok C. S. Loke Y. K. Hale R. Potter J. F. Myint P. K. (2011). Atrial fibrillation and incidence of dementia: a systematic review and meta-analysis. Neurology 76, 914–922. 10.1212/WNL.0b013e31820f2e38 21383328

[B126] Lahiri D. K. Utsuki T. Chen D. Farlow M. R. Shoaib M. Ingram D. K.. (2002). Nicotine reduces the secretion of Alzheimer’s β-amyloid precursor protein containing β-amyloid peptide in the rat without altering synaptic proteins. Ann. N Y Acad. Sci. 965, 364–372. 10.1111/j.1749-6632.2002.tb04178.x 12105112

[B127] Langbaum J. B. S. Chen K. Launer L. J. Fleisher A. S. Lee W. Liu X.. (2012). Blood pressure is associated with higher brain amyloid burden and lower glucose metabolism in healthy late middle-age persons. Neurobiol. Aging 33, 827.e11–827.e19. 10.1016/j.neurobiolaging.2011.06.020 21821316 PMC3236809

[B128] Launer L. J. Ross G. W. Petrovitch H. Masaki K. Foley D. White L. R.. (2000). Midlife blood pressure and dementia: the honolulu-asia aging study. Neurobiol. Aging 21, 49–55. 10.1016/S0197-4580(00)00096-8 10794848

[B129] Law L. L. Sprecher K. E. Dougherty R. J. Edwards D. F. Koscik R. L. Gallagher C. L.. (2019). Cardiorespiratory fitness modifies influence of sleep problems on cerebrospinal fluid biomarkers in an at-risk cohort. J. Alzheimers Dis. 69, 111–121. 10.3233/JAD-180291 30958346 PMC6675618

[B130] Lee P. N. (1994). Smoking and Alzheimer’s disease: a review of the epidemiological evidence. Neuroepidemiology 13, 131–144. 10.1159/000110372 8090255

[B131] Leem Y. H. Lim H. J. Shim S. B. Cho J. Y. Kim B. S. Han P. L. (2009). Repression of tau hyperphosphorylation by chronic endurance exercise in aged transgenic mouse model of tauopathies. J. Neurosci. Res. 87, 2561–2570. 10.1002/jnr.22075 19360903

[B134] Li P. Hsiao I. T. Liu C. Y. Chen C. H. Huang S. Y. Yen T. C.. (2017). β-amyloid deposition in patients with major depressive disorder with differing levels of treatment resistance: a pilot study. EJNMMI Res. 7:24. 10.1186/s13550-017-0273-4 28324341 PMC5360749

[B132] Li G. Mayer C. L. Morelli D. Millard S. P. Raskind W. H. Petrie E. C.. (2017). Effect of simvastatin on CSF Alzheimer disease biomarkers in cognitively normal adults. Neurology 89, 1251–1255. 10.1212/WNL.0000000000004392 28821686 PMC5606918

[B133] Li J. Wang Y. J. Zhang M. Xu Z. Q. Gao C. Y. Fang C. Q.. (2011). Vascular risk factors promote conversion from mild cognitive impairment to Alzheimer disease. Neurology 76, 1485–1491. 10.1212/WNL.0b013e318217e7a4 21490316

[B135] Li X. Song D. Leng S. X. (2015). Link between type 2 diabetes and Alzheimer’s disease: from epidemiology to mechanism and treatment. Clin. Interv. Aging 10, 549–560. 10.2147/CIA.S74042 25792818 PMC4360697

[B138] Liu Y. Liu F. Grundke-Iqbal I. Iqbal K. Gong C. X. (2009). Brain glucose transporters, O-GlcNAcylation and phosphorylation of tau in diabetes and Alzheimer’s disease. J. Neurochem. 111, 242–249. 10.1111/j.1471-4159.2009.06320.x 19659459 PMC2760012

[B139] Liu Y. Liu F. Iqbal K. Grundke-Iqbal I. Gong C. X. (2008). Decreased glucose transporters correlate to abnormal hyperphosphorylation of tau in Alzheimer disease. FEBS Lett. 582, 359–364. 10.1016/j.febslet.2007.12.035 18174027 PMC2247364

[B137] Liu H. Zhao G. Cai K. Zhao H. Shi L. (2011). Treadmill exercise prevents decline in spatial learning and memory in APP/PS1 transgenic mice through improvement of hippocampal long-term potentiation. Behav. Brain Res. 218, 308–314. 10.1016/j.bbr.2010.12.030 21192984

[B136] Liu H.-L. Zhao G. Zhang H. Shi L. D. (2013). Long-term treadmill exercise inhibits the progression of Alzheimer’s disease-like neuropathology in the hippocampus of APP/PS1 transgenic mice. Behav. Brain Res. 256, 261–272. 10.1016/j.bbr.2013.08.008 23968591

[B140] Livingston G. Sommerlad A. Orgeta V. Costafreda S. G. Huntley J. Ames D.. (2017). Dementia prevention, intervention and care. Lancet 390, 2673–2734. 10.1016/S0140-6736(17)31363-6 28735855

[B141] Loewenstein R. J. Weingartner H. Gillin J. C. Kaye W. Ebert M. Mendelson W. B. (1982). Disturbances of sleep and cognitive functioning in patients with dementia. Neurobiol. Aging 3, 371–377. 10.1016/0197-4580(82)90025-2 7170053

[B142] Loughrey D. G. Lavecchia S. Brennan S. Lawlor B. A. Kelly M. E. (2017). The impact of the mediterranean diet on the cognitive functioning of healthy older adults: a systematic review and meta-analysis. Adv. Nutr. 8, 571–586. 10.3945/an.117.015495 28710144 PMC5502874

[B143] Lourenco M. V. Frozza R. L. de Freitas G. B. Zhang H. Kincheski G. C. Ribeiro F. C.. (2019). Exercise-linked FNDC5/irisin rescues synaptic plasticity and memory defects in Alzheimer’s models. Nat. Med. 25, 165–175. 10.1038/s41591-018-0275-4 30617325 PMC6327967

[B144] Love S. Miners J. S. (2016). Cerebrovascular disease in ageing and Alzheimer’s disease. Acta Neuropathol. 131, 645–658. 10.1007/s00401-015-1522-0 26711459 PMC4835514

[B145] Lu Y. Dong Y. Tucker D. Wang R. Ahmed M. E. Brann D.. (2017). Treadmill exercise exerts neuroprotection and regulates microglial polarization and oxidative stress in a streptozotocin-induced rat model of sporadic Alzheimer’s disease. J. Alzheimers Dis. 56, 1469–1484. 10.3233/JAD-160869 28157094 PMC5450951

[B146] Luchsinger J. A. Gustafson D. R. (2009). Adiposity, type 2 diabetes and Alzheimer’s disease. J. Alzheimers Dis. 16, 693–704. 10.3233/JAD-2009-1022 19387106 PMC2705908

[B147] Luchsinger J. A. Reitz C. Honig L. S. Tang M. X. Shea S. Mayeux R. (2005). Aggregation of vascular risk factors and risk of incident Alzheimer disease. Neurology 65, 545–551. 10.1212/01.WNL.0000172914.08967.dc 16116114 PMC1619350

[B148] Luchsinger J. A. Tang M.-X. Siddiqui M. Shea S. Mayeux R. (2004). Alcohol intake and risk of dementia. J. Am. Geriatr. Soc. 52, 540–546. 10.1111/j.1532-5415.2004.52159.x 15066068

[B149] Lundgaard I. Wang W. Eberhardt A. Vinitsky H. S. Reeves B. C. Peng S.. (2018). Beneficial effects of low alcohol exposure, but adverse effects of high alcohol intake on glymphatic function. Sci. Rep. 8:2246. 10.1038/s41598-018-20424-y 29396480 PMC5797082

[B150] Lyketsos C. G. Tune L. E. Pearlson G. Steele C. (1996). Major depression in Alzheimer’s disease: an interaction between gender and family history. Psychosomatics 37, 380–384. 10.1016/S0033-3182(96)71552-9 8701017

[B151] Magariños A. M. Orchinik M. McEwen B. S. (1998). Morphological changes in the hippocampal CA3 region induced by non-invasive glucocorticoid administration: a paradox. Brain Res. 809, 314–318. 10.1016/s0006-8993(98)00882-8 9853126

[B152] Maheshwari A. Marks R. L. Yu K. M. Noebels J. L. (2016). Shift in interictal relative γ power as a novel biomarker for drug response in two mouse models of absence epilepsy. Epilepsia 57, 79–88. 10.1111/epi.13265 26663261 PMC5551895

[B153] McCleery J. Cohen D. A. Sharpley A. L. (2016). Pharmacotherapies for sleep disturbances in dementia. Cochrane Database Syst. Rev. 11:CD009178. 10.1002/14651858.CD009178.pub3 27851868 PMC6464889

[B154] Mebane-Sims I. Alzheimer’s Association (2009). 2009 Alzheimer’s disease facts and figures. Alzheimers Dement. 5, 234–270. 10.1016/j.jalz.2009.03.001 19426951

[B155] Mehta D. Jackson R. Paul G. Shi J. Sabbagh M. (2017). Why do trials for Alzheimer’s disease drugs keep failing? A discontinued drug perspective for 2010–2015. Expert Opin. Investig. Drugs 26, 735–739. 10.1080/13543784.2017.1323868 28460541 PMC5576861

[B156] Miklossy J. Qing H. Radenovic A. Kis A. Vileno B. Làszló F.. (2010). β amyloid and hyperphosphorylated tau deposits in the pancreas in type 2 diabetes. Neurobiol. Aging 31, 1503–1515. 10.1016/j.neurobiolaging.2008.08.019 18950899 PMC4140193

[B157] Monroe S. M. Slavich G. M. Torres L. D. Gotlib I. H. (2007). Major life events and major chronic difficulties are differentially associated with history of major depressive episodes. J. Abnorm. Psychol. 116, 116–124. 10.1037/0021-843X.116.1.116 17324022 PMC3631311

[B158] Monti G. Tondelli M. Giovannini G. Bedin R. Nichelli P. F. Trenti T.. (2015). Cerebrospinal fluid tau proteins in status epilepticus. Epilepsy Behav. 49, 150–154. 10.1016/j.yebeh.2015.04.030 25958230

[B159] Morales R. Moreno-Gonzalez I. Soto C. (2013). Cross-seeding of misfolded proteins: implications for etiology and pathogenesis of protein misfolding diseases. PLoS Pathog. 9:e1003537. 10.1371/journal.ppat.1003537 24068917 PMC3777858

[B160] Moran M. Lynch C. Walsh C. Coen R. Coakley D. Lawlor B. (2005). Sleep disturbance in mild to moderate Alzheimer’s disease. Sleep Med. 6, 347–352. 10.1016/j.sleep.2004.12.005 15978517

[B162] Moreno-Gonzalez I. Baglietto-Vargas D. Sanchez-Varo R. Jimenez S. Trujillo-Estrada L. Sanchez-Mejias E.. (2009). Extracellular amyloid-β and cytotoxic glial activation induce significant entorhinal neuron loss in young PS1M146L/APP751SL mice. J. Alzheimers Dis. 18, 755–776. 10.3233/JAD-2009-1192 19661615

[B163] Moreno-Gonzalez I. Edwards Iii G. Salvadores N. Shahnawaz M. Diaz-Espinoza R. Soto C. (2017). Molecular interaction between type 2 diabetes and Alzheimer’s disease through cross-seeding of protein misfolding. Mol. Psychiatry 22, 1327–1334. 10.1038/mp.2016.230 28044060 PMC5495631

[B164] Moreno-Gonzalez I. Estrada L. D. Sanchez-Mejias E. Soto C. (2013). Smoking exacerbates amyloid pathology in a mouse model of Alzheimer’s disease. Nat. Commun. 4:1495. 10.1038/ncomms2494 23422663

[B161] Moreno-Gonzalez I. Soto C. (2011). Misfolded protein aggregates: mechanisms, structures and potential for disease transmission. Semin. Cell Dev. Biol. 22, 482–487. 10.1016/j.semcdb.2011.04.002 21571086 PMC3175247

[B166] Morris M. C. Tangney C. C. Wang Y. Sacks F. M. Bennett D. A. Aggarwal N. T. (2015). MIND diet associated with reduced incidence of Alzheimer’s disease. Alzheimers Dement. 11, 1007–1014. 10.1016/j.jalz.2014.11.009 25681666 PMC4532650

[B165] Morris J. K. Vidoni E. D. Johnson D. K. Van Sciver A. Mahnken J. D. Honea R. A.. (2017). Aerobic exercise for Alzheimer’s disease: a randomized controlled pilot trial. PLoS One 12:e0170547. 10.1371/journal.pone.0170547 28187125 PMC5302785

[B167] Mucke L. Selkoe D. J. (2012). Neurotoxicity of amyloid β-protein: synaptic and network dysfunction. Cold Spring Harb. Perspect. Med. 2:a006338. 10.1101/cshperspect.a006338 22762015 PMC3385944

[B168] Muñoz G. Urrutia J. C. Burgos C. F. Silva V. Aguilar F. Sama M.. (2015). Low concentrations of ethanol protect against synaptotoxicity induced by Aβ in hippocampal neurons. Neurobiol. Aging 36, 845–856. 10.1016/j.neurobiolaging.2014.10.017 25433458

[B169] Muqtadar H. Testai F. D. Gorelick P. B. (2012). The dementia of cardiac disease. Curr. Cardiol. Rep. 14, 732–740. 10.1007/s11886-012-0304-8 22968344

[B170] Neeper S. A. Góauctemez-Pinilla F. Choi J. Cotman C. (1995). Exercise and brain neurotrophins. Nature 373:109. 10.1038/373109a0 7816089

[B171] Nelson R. Guo Z. Halagappa V. Pearson M. Gray A. Matsuoka Y.. (2007). Prophylactic treatment with paroxetine ameliorates behavioral deficits and retards the development of amyloid and tau pathologies in 3xTgAD mice. Exp. Neurol. 205, 166–176. 10.1016/j.expneurol.2007.01.037 17368447 PMC1979096

[B172] Ngandu T. Lehtisalo J. Solomon A. Levälahti E. Ahtiluoto S. Antikainen R.. (2015). A 2 year multidomain intervention of diet, exercise, cognitive training and vascular risk monitoring versus control to prevent cognitive decline in at-risk elderly people (FINGER): a randomised controlled trial. Lancet 385, 2255–2263. 10.1016/S0140-6736(15)60461-5 25771249

[B174] Nichol K. Deeny S. P. Seif J. Camaclang K. Cotman C. W. (2009). Exercise improves cognition and hippocampal plasticity in APOE ε4 mice. Alzheimers Dement. 5, 287–294. 10.1016/j.jalz.2009.02.006 19560099 PMC4105011

[B173] Nichol K. E. Parachikova A. I. Cotman C. W. (2007). Three weeks of running wheel exposure improves cognitive performance in the aged Tg2576 mouse. Behav. Brain Res. 184, 124–132. 10.1016/j.bbr.2007.06.027 17698211 PMC2215770

[B175] Nigam S. M. Xu S. Kritikou J. S. Marosi K. Brodin L. Mattson M. P. (2017). Exercise and BDNF reduce Aβ production by enhancing α-secretase processing of APP. J. Neurochem. 142, 286–296. 10.1111/jnc.14034 28382744 PMC5498234

[B176] Ninomiya T. Ohara T. Hirakawa Y. Yoshida D. Doi Y. Hata J.. (2011). Midlife and late-life blood pressure and dementia in japanese elderly: the hisayama study. Hypertension 58, 22–28. 10.1161/hypertensionaha.110.163055 21555680

[B177] Nobili A. Latagliata E. C. Viscomi M. T. Cavallucci V. Cutuli D. Giacovazzo G.. (2017). Dopamine neuronal loss contributes to memory and reward dysfunction in a model of Alzheimer’s disease. Nat. Commun. 8:14727. 10.1038/ncomms14727 28367951 PMC5382255

[B178] Nordberg A. (2015). Towards early diagnosis in Alzheimer disease. Nat. Rev. Neurol. 11, 69–70. 10.1038/nrneurol.2014.257 25623789

[B179] Nordberg A. Hellström-Lindahl E. Lee M. Johnson M. Mousavi M. Hall R.. (2002). Chronic nicotine treatment reduces β-amyloidosis in the brain of a mouse model of Alzheimer’s disease (APPsw). J. Neurochem. 81, 655–658. 10.1046/j.1471-4159.2002.00874.x 12065674

[B180] Nordström A. Nordström P. (2018). Traumatic brain injury and the risk of dementia diagnosis: a nationwide cohort study. PLoS Med. 15:e1002496. 10.1371/journal.pmed.1002496 29381704 PMC5790223

[B181] Oddo S. Caccamo A. Green K. N. Liang K. Tran L. Chen Y.. (2005). Chronic nicotine administration exacerbates tau pathology in a transgenic model of Alzheimer’s disease. Proc. Natl. Acad. Sci. U S A 102, 3046–3051. 10.1073/pnas.0408500102 15705720 PMC549455

[B182] Ohia-Nwoko O. Montazari S. Lau Y. S. Eriksen J. L. (2014). Long-term treadmill exercise attenuates tau pathology in P301S tau transgenic mice. Mol. Neurodegener. 9:54. 10.1186/1750-1326-9-54 25432085 PMC4280713

[B183] Omar S. H. Scott C. J. Hamlin A. S. Obied H. K. (2018). Biophenols: Enzymes (β-secretase, Cholinesterases, histone deacetylase and tyrosinase) inhibitors from olive (*Olea europaea L.*). Fitoterapia 128, 118–129. 10.1016/j.fitote.2018.05.011 29772299

[B184] Ott A. Breteler M. M. B. De Bruyne M. C. Van Harskamp F. Grobbee D. E. Hofman A. (1997). Atrial fibrillation and dementia in a population-based study: the Rotterdam study. Stroke 28, 316–321. 10.1161/01.STR.28.2.316 9040682

[B185] Ott A. Slooter A. J. Hofman A. van Harskamp F. Witteman J. C. Van Broeckhoven C.. (1998). Smoking and risk of dementia and Alzheimer’s disease in a population-based cohort study: the Rotterdam Study. Lancet 351, 1840–1843. 10.1016/s0140-6736(97)07541-7 9652667

[B186] Ott A. Stolk R. P. van Harskamp F. Pols H. A. Hofman A. Breteler M. M. (1999). Diabetes mellitus and the risk of dementia: the Rotterdam study. Neurology 53, 1937–1942. 10.1212/wnl.53.9.1937 10599761

[B187] Panpalli Ates M. Karaman Y. Guntekin S. Ergun M. A. (2016). Analysis of genetics and risk factors of Alzheimer’s disease. Neuroscience 325, 124–131. 10.1016/j.neuroscience.2016.03.051 27026590

[B188] Parrott M. D. Winocur G. Bazinet R. P. Ma D. W. L. Greenwood C. E. (2015). Whole-food diet worsened cognitive dysfunction in an Alzheimer’s disease mouse model. Neurobiol. Aging 36, 90–99. 10.1016/j.neurobiolaging.2014.08.013 25212462

[B190] Pendlebury S. T. Rothwell P. M. (2009). Prevalence, incidence and factors associated with pre-stroke and post-stroke dementia: a systematic review and meta-analysis. Lancet Neurol. 8, 1006–1018. 10.1016/s1474-4422(09)70236-4 19782001

[B191] Peng Y. Hou C. Yang Z. Li C. Jia L. Liu J.. (2016). Hydroxytyrosol mildly improve cognitive function independent of APP processing in APP/PS1 mice. Mol. Nutr. Food Res. 60, 2331–2342. 10.1002/mnfr.201600332 27287957

[B192] Peters R. Beckett N. Forette F. Tuomilehto J. Clarke R. Ritchie C.. (2008). Incident dementia and blood pressure lowering in the Hypertension in the Very Elderly Trial cognitive function assessment (HYVET-COG): a double-blind, placebo controlled trial. Lancet Neurol. 7, 683–689. 10.1016/S1474-4422(08)70143-1 18614402

[B193] Petrovitch H. White L. R. Izmirilian G. Ross G. W. Havlik R. J. Markesbery W.. (2000). Midlife blood pressure and neuritic plaques, neurofibrillary tangles, and brain weight at death: the HAAS. Neurobiol. Aging 21, 57–62. 10.1016/s0197-4580(00)00106-8 10794849

[B194] Pfeifer L. A. White L. R. Ross G. W. Petrovitch H. Launer L. J. (2002). Cerebral amyloid angiopathy and cognitive function: the HAAS autopsy study. Neurology 58, 1629–1634. 10.1212/wnl.58.11.1629 12058090

[B195] Piazza-Gardner A. K. Gaffud T. J. B. Barry A. E. (2013). The impact of alcohol on Alzheimer’s disease: a systematic review. Aging Ment. Health 17, 133–146. 10.1080/13607863.2012.742488 23171229

[B196] Pitkälä K. H. Pöysti M. M. Laakkonen M. L. Tilvis R. S. Savikko N. Kautiainen H.. (2013). Effects of the Finnish Alzheimer Disease Exercise Trial (FINALEX): a randomized controlled trial. JAMA Intern. Med. 173, 894–901. 10.1001/jamainternmed.2013.359 23589097

[B197] Plassman B. L. Havlik R. J. Steffens D. C. Helms M. J. Newman T. N. Drosdick D.. (2000). Documented head injury in early adulthood and risk of Alzheimer’s disease and other dementias. Neurology 55, 1158–1166. 10.1212/wnl.55.8.1158 11071494

[B198] Querfurth H. W. LaFerla F. M. (2010). Alzheimer’s disease. N. Engl. J. Med. 362, 329–344. 10.1056/NEJMra0909142 20107219

[B199] Rabin J. S. Yang H. Schultz A. P. Hanseeuw B. J. Hedden T. Viswanathan A.. (2019). Vascular risk and β-amyloid are synergistically associated with cortical tau. Ann. Neurol. 85, 272–279. 10.1002/ana.25399 30565287 PMC6351182

[B200] Ramos B. Baglietto-Vargas D. del Rio J. C. Moreno-Gonzalez I. Santa-Maria C. Jimenez S.. (2006). Early neuropathology of somatostatin/NPY GABAergic cells in the hippocampus of a PS1 × APP transgenic model of Alzheimer’s disease. Neurobiol. Aging 27, 1658–1672. 10.1016/j.neurobiolaging.2005.09.022 16271420

[B201] Raschetti R. Albanese E. Vanacore N. Maggini M. (2007). Cholinesterase inhibitors in mild cognitive impairment: a systematic review of randomised trials. PLoS Med. 4:e338. 10.1371/journal.pmed.0040338 18044984 PMC2082649

[B202] Rasmuson S. Andrew R. Näsman B. Seckl J. R. Walker B. R. Olsson T. (2001). Increased glucocorticoid production and altered cortisol metabolism in women with mild to moderate Alzheimer’s disease. Biol. Psychiatry 49, 547–552. 10.1016/s0006-3223(00)01015-5 11257240

[B203] Rehm J. (2011). The risks associated with alcohol use and alcoholism. Alcohol Res. Health 34, 135–143. 22330211 PMC3307043

[B204] Reijs B. L. R. Vos S. J. B. Soininen H. Lötjonen J. Koikkalainen J. Pikkarainen M.. (2017). Association between later life lifestyle factors and Alzheimer’s disease biomarkers in non-demented individuals: a longitudinal descriptive cohort study. J. Alzheimers Dis. 60, 1387–1395. 10.3233/JAD-170039 29036813

[B205] Reitz C. den Heijer T. van Duijn C. Hofman A. Breteler M. M. B. (2007). Relation between smoking and risk of dementia and Alzheimer disease: the Rotterdam study. Neurology 69, 998–1005. 10.1212/01.wnl.0000271395.29695.9a 17785668

[B207] Richards M. Jarvis M. J. Thompson N. Wadsworth M. E. J. (2003). Cigarette smoking and cognitive decline in midlife: evidence from a prospective birth cohort study. Am. J. Public Health 93, 994–998. 10.2105/ajph.93.6.994 12773367 PMC1447882

[B208] Rivera E. J. Goldin A. Fulmer N. Tavares R. Wands J. R. de la Monte S. M. (2005). Insulin and insulin-like growth factor expression and function deteriorate with progression of Alzheimer’s disease: link to brain reductions in acetylcholine. J. Alzheimers Dis. 8, 247–268. 10.3233/jad-2005-8304 16340083

[B209] Roberson E. D. Halabisky B. Yoo J. W. Yao J. Chin J. Yan F.. (2011). Amyloid-β/Fyn-induced synaptic, network, and cognitive impairments depend on tau levels in multiple mouse models of Alzheimer’s disease. J. Neurosci. 31, 700–711. 10.1523/jneurosci.4152-10.2011 21228179 PMC3325794

[B210] Roh J. H. Huang Y. Bero A. W. Kasten T. Stewart F. R. Bateman R. J.. (2012). Disruption of the sleep-wake cycle and diurnal fluctuation of amyloid-β in mice with Alzheimer’s disease pathology. Sci. Transl. Med. 4:150ra122. 10.1126/scitranslmed.3004291 22956200 PMC3654377

[B211] Roh J. H. Jiang H. Finn M. B. Stewart F. R. Mahan T. E. Cirrito J. R. (2015). Potential role of orexin and sleep modulation in the pathogenesis of Alzheimer’s disease. J. Exp. Med. 212:121 10.1084/jem.2014178812122014c PMC4267230 25422493

[B212] Roher A. E. Esh C. Kokjohn T. A. Kalback W. Luehrs D. C. Seward J. D.. (2003). Circle of willis atherosclerosis is a risk factor for sporadic Alzheimer’s disease. Arterioscler. Thromb. Vasc. Biol. 23, 2055–2062. 10.1161/01.atv.0000095973.42032.44 14512367

[B213] Rolland Y. Pillard F. Klapouszczak A. Reynish E. Thomas D. Andrieu S.. (2007). Exercise program for nursing home residents with Alzheimer’s disease: a 1-year randomized, controlled trial. J. Am. Geriatr. Soc. 55, 158–165. 10.1111/j.1532-5415.2007.01035.x 17302650

[B214] Ross J. A. Gliebus G. Van Bockstaele E. J. (2018). Stress induced neural reorganization: a conceptual framework linking depression and Alzheimer’s disease. Prog. Neuropsychopharmacol. Biol. Psychiatry 85, 136–151. 10.1016/j.pnpbp.2017.08.004 28803923 PMC5809232

[B215] Russo A. Palumbo M. Aliano C. Lempereur L. Scoto G. Renis M. (2003). Red wine micronutrients as protective agents in Alzheimer-like induced insult. Life Sci. 72, 2369–2379. 10.1016/s0024-3205(03)00123-1 12639702

[B216] Sah S. K. Lee C. Jang J.-H. Park G. H. (2017). Effect of high-fat diet on cognitive impairment in triple-transgenic mice model of Alzheimer’s disease. Biochem. Biophys. Res. Commun. 493, 731–736. 10.1016/j.bbrc.2017.08.122 28865961

[B217] Sanchez P. E. Zhu L. Verret L. Vossel K. A. Orr A. G. Cirrito J. R.. (2012). Levetiracetam suppresses neuronal network dysfunction and reverses synaptic and cognitive deficits in an Alzheimer’s disease model. Proc. Natl. Acad. Sci. U S A 109, E2895–E2903. 10.1073/pnas.1121081109 22869752 PMC3479491

[B218] Scarmeas N. Luchsinger J. A. Mayeux R. Stern Y. (2007). Mediterranean diet and Alzheimer disease mortality. Neurology 69, 1084–1093. 10.1212/01.wnl.0000277320.50685.7c 17846408 PMC2673956

[B93] Scharfman H. E. (2007). The neurobiology of epilepsy. Curr. Neurol. Neurosci. Rep. 7, 348–354. 10.1007/s11910-007-0053-z 17618543 PMC2492886

[B219] Shakour N. Bianconi V. Pirro M. Barreto G. E. Hadizadeh F. Sahebkar A. (2019). In silico evidence of direct interaction between statins and β-amyloid. J. Cell. Biochem. 120, 4710–4715. 10.1002/jcb.27761 30260016

[B220] Shen Y. Joachimiak A. Rich Rosner M. Tang W. J. (2006). Structures of human insulin-degrading enzyme reveal a new substrate recognition mechanism. Nature 443, 870–874. 10.1038/nature05143 17051221 PMC3366509

[B221] Shokri-Kojori E. Wang G.-J. Wiers C. E. Demiral S. B. Guo M. Kim S. W.. (2018). β-Amyloid accumulation in the human brain after one night of sleep deprivation. Proc. Natl. Acad. Sci. U S A 115, 4483–4488. 10.1073/pnas.1721694115 29632177 PMC5924922

[B222] Sim Y.-J. (2014). Treadmill exercise alleviates impairment of spatial learning ability through enhancing cell proliferation in the streptozotocin-induced Alzheimer’s disease rats. J. Exerc. Rehabil. 10, 81–88. 10.12965/jer.140102 24877042 PMC4025554

[B223] Sims-Robinson C. Kim B. Rosko A. Feldman E. L. (2010). How does diabetes accelerate Alzheimer disease pathology? Nat. Rev. Neurol. 6, 551–559. 10.1038/nrneurol.2010.130 20842183 PMC3199576

[B224] Smith C. Graham D. I. Murray L. S. Nicoll J. A. R. (2003). Tau immunohistochemistry in acute brain injury. Neuropathol. Appl. Neurobiol. 29, 496–502. 10.1046/j.1365-2990.2003.00488.x 14507341

[B225] Soininen H. Solomon A. Visser P. J. Hendrix S. B. Blennow K. Kivipelto M.. (2017). 24-month intervention with a specific multinutrient in people with prodromal Alzheimer’s disease (LipiDiDiet): a randomised, double-blind, controlled trial. Lancet Neurol. 16, 965–975. 10.1016/S1474-4422(17)30332-0 29097166 PMC5697936

[B226] Soto C. (2003). Unfolding the role of protein misfolding in neurodegenerative diseases. Nat. Rev. Neurosci. 4, 49–60. 10.1038/nrn1007 12511861

[B227] Steenland K. Karnes C. Seals R. Carnevale C. Hermida A. Levey A. (2012). Late-life depression as a risk factor for mild cognitive impairment or Alzheimer’s disease in 30 us Alzheimer’s disease centers. J. Alzheimers Dis. 31, 265–275. 10.3233/JAD-2012-111922 22543846 PMC3729228

[B229] Sun X. He G. Qing H. Zhou W. Dobie F. Cai F.. (2006). Hypoxia facilitates Alzheimer’s disease pathogenesis by up-regulating BACE1 gene expression. Proc. Natl. Acad. Sci. U S A 103, 18727–18732. 10.1073/pnas.0606298103 17121991 PMC1693730

[B228] Sun M.-K. Hongpaisan J. Nelson T. J. Alkon D. L. (2008). Poststroke neuronal rescue and synaptogenesis mediated *in vivo* by protein kinase C in adult brains. Proc. Natl. Acad. Sci. U S A 105, 13620–13625. 10.1073/pnas.0805952105 18768786 PMC2533239

[B230] Swan G. E. Lessov-Schlaggar C. N. (2007). The effects of tobacco smoke and nicotine on cognition and the brain. Neuropsychol. Rev. 17, 259–273. 10.1007/s11065-007-9035-9 17690985

[B231] Taillard J. Sagaspe P. Berthomier C. Brandewinder M. Amieva H. Dartigues J.-F.. (2019). Non-REM sleep characteristics predict early cognitive impairment in an aging population. Front. Neurol. 10:197. 10.3389/fneur.2019.00197 30918496 PMC6424890

[B232] Tangney C. C. Kwasny M. J. Li H. Wilson R. S. Evans D. A. Morris M. C. (2011). Adherence to a Mediterranean-type dietary pattern and cognitive decline in a community population. Am. J. Clin. Nutr. 93, 601–607. 10.3945/ajcn.110.007369 21177796 PMC3041601

[B234] Taylor W. D. McQuoid D. R. Payne M. E. Zannas A. S. MacFall J. R. Steffens D. C. (2014). Hippocampus atrophy and the longitudinal course of late-life depression. Am. J. Geriatr. Psychiatry 22, 1504–1512. 10.1016/j.jagp.2013.11.004 24378256 PMC4031313

[B233] Taylor M. K. Sullivan D. K. Swerdlow R. H. Vidoni E. D. Morris J. K. Mahnken J. D.. (2017). A high-glycemic diet is associated with cerebral amyloid burden in cognitively normal older adults. Am. J. Clin. Nutr. 106, 1463–1470. 10.3945/ajcn.117.162263 29070566 PMC5698843

[B235] Thériault P. ElAli A. Rivest S. (2016). High fat diet exacerbates Alzheimer’s disease-related pathology in APPswe/PS1 mice. Oncotarget 7, 67808–67827. 10.18632/oncotarget.12179 27661129 PMC5356521

[B236] Toda A. Tagata Y. Nakada T. Komatsu M. Shibata N. Arai H. (2013). Changes in mini-mental state examination score in Alzheimer’s disease patients after stopping habitual drinking. Psychogeriatrics 13, 94–98. 10.1111/psyg.12008 23909966

[B237] Toledo J. B. Arnold S. E. Raible K. Brettschneider J. Xie S. X. Grossman M.. (2013). Contribution of cerebrovascular disease in autopsy confirmed neurodegenerative disease cases in the National Alzheimer’s Coordinating Centre. Brain 136, 2697–2706. 10.1093/brain/awt188 23842566 PMC3858112

[B238] Topiwala A. Allan C. L. Valkanova V. Zsoldos E. Filippini N. Sexton C.. (2017). Moderate alcohol consumption as risk factor for adverse brain outcomes and cognitive decline: longitudinal cohort study. BMJ 357:j2353. 10.1136/bmj.j2353 28588063 PMC5460586

[B239] Trichopoulou A. Kyrozis A. Rossi M. Katsoulis M. Trichopoulos D. La Vecchia C.. (2015). Mediterranean diet and cognitive decline over time in an elderly Mediterranean population. Eur. J. Nutr. 54, 1311–1321. 10.1007/s00394-014-0811-z 25482573

[B240] Tseng B. Y. Uh J. Rossetti H. C. Cullum C. M. Diaz-Arrastia R. F. Levine B. D.. (2013). Masters athletes exhibit larger regional brain volume and better cognitive performance than sedentary older adults. J. Magn. Reson. Imaging 38, 1169–1176. 10.1002/jmri.24085 23908143 PMC3812419

[B241] Um H. S. Kang E. B. Leem Y. H. Cho I. H. Yang C. H. Chae K. R.. (2008). Exercise training acts as a therapeutic strategy for reduction of the pathogenic phenotypes for Alzheimer’s disease in an NSE/APPSw-transgenic model. Int. J. Mol. Med. 22, 529–539. 10.3892/ijmm_00000052 18813861

[B242] Uryu K. Chen X.-H. Martinez D. Browne K. D. Johnson V. E. Graham D. I.. (2007). Multiple proteins implicated in neurodegenerative diseases accumulate in axons after brain trauma in humans. Exp. Neurol. 208, 185–192. 10.1016/j.expneurol.2007.06.018 17826768 PMC3979356

[B243] Valente T. Gella A. Fernàndez-Busquets X. Unzeta M. Durany N. (2010). Immunohistochemical analysis of human brain suggests pathological synergism of Alzheimer’s disease and diabetes mellitus. Neurobiol. Dis. 37, 67–76. 10.1016/j.nbd.2009.09.008 19778613

[B244] Valls-Pedret C. Sala-Vila A. Serra-Mir M. Corella D. de la Torre R. Martínez-González M. Á.. (2015). Mediterranean diet and age-related cognitive decline: a randomized clinical trial. JAMA Intern. Med. 175, 1094–1103. 10.1001/jamainternmed.2015.1668 25961184

[B245] van Dyck C. H. (2018). Anti-amyloid-β monoclonal antibodies for Alzheimer’s disease: pitfalls and promise. Biol. Psychiatry 83, 311–319. 10.1016/j.biopsych.2017.08.010 28967385 PMC5767539

[B246] Vemuri P. Lesnick T. G. Przybelski S. A. Knopman D. S. Lowe V. J. Graff-Radford J.. (2017). Age, vascular health, and Alzheimer disease biomarkers in an elderly sample. Ann. Neurol. 82, 706–718. 10.1002/ana.25071 29023983 PMC5696029

[B247] Vetreno R. P. Crews F. T. (2018). Adolescent binge ethanol-induced loss of basal forebrain cholinergic neurons and neuroimmune activation are prevented by exercise and indomethacin. PLoS One 13:e0204500. 10.1371/journal.pone.0204500 30296276 PMC6175501

[B248] Vidoni E. D. Van Sciver A. Johnson D. K. He J. Honea R. Haines B.. (2012). A community-based approach to trials of aerobic exercise in aging and Alzheimer’s disease. Contemp. Clin. Trials 33, 1105–1116. 10.1016/j.cct.2012.08.002 22903151 PMC3468654

[B249] Vijayan M. Reddy P. H. (2016). Stroke, vascular dementia, and Alzheimer’s disease: molecular links. J. Alzheimers Dis. 54, 427–443. 10.3233/JAD-160527 27567871 PMC5793908

[B250] Vossel K. A. Beagle A. J. Rabinovici G. D. Shu H. Lee S. E. Naasan G.. (2013). Seizures and epileptiform activity in the early stages of Alzheimer disease. JAMA Neurol. 70, 1158–1166. 10.1001/jamaneurol.2013.136 23835471 PMC4013391

[B251] Vossel K. A. Ranasinghe K. G. Beagle A. J. Mizuiri D. Honma S. M. Dowling A. F.. (2016). Incidence and impact of subclinical epileptiform activity in Alzheimer’s disease. Ann. Neurol. 80, 858–870. 10.1002/ana.24794 27696483 PMC5177487

[B189] Wang J. Ho L. Zhao Z. Seror I. Humala N. Dickstein D. L.. (2006). Moderate consumption of Cabernet Sauvignon attenuates Aβ neuropathology in a mouse model of Alzheimer’s disease. FASEB J. 20, 2313–2320. 10.1096/fj.06-6281com 17077308

[B252] Weissenborn R. Duka T. (2003). Acute alcohol effects on cognitive function in social drinkers: their relationship to drinking habits. Psychopharmacology 165, 306–312. 10.1007/s00213-002-1281-1 12439627

[B253] Westermark P. (2011). Amyloid in the islets of Langerhans: thoughts and some historical aspects. Ups. J. Med. Sci. 116, 81–89. 10.3109/03009734.2011.573884 21486192 PMC3078536

[B254] Westwood A. J. Beiser A. Jain N. Himali J. J. DeCarli C. Auerbach S. H.. (2017). Prolonged sleep duration as a marker of early neurodegeneration predicting incident dementia. Neurology 88, 1172–1179. 10.1212/WNL.0000000000003732 28228567 PMC5373785

[B255] Winter B. Breitenstein C. Mooren F. C. Voelker K. Fobker M. Lechtermann A.. (2007). High impact running improves learning. Neurobiol. Learn. Mem. 87, 597–609. 10.1016/j.nlm.2006.11.003 17185007

[B256] World Health Organization (2013). WHO Report on the Global Tobacco Epidemic. Enforcing Bans on Tobacco Advertising, Promotion and Sponsorship Fresh and Alive Mpower. Includes A Special Section on Five Years of Progress. Geneva: World Health Organization.

[B257] World Health Organization (2017). Depression and Other Common Mental Disorders: Global Health Estimates. Geneva: World Health Organization.

[B258] Wrann C. D. White J. P. Salogiannnis J. Laznik-Bogoslavski D. Wu J. Ma D.. (2013). Exercise induces hippocampal BDNF through a PGC-1α/FNDC5 pathway. Cell Metab. 18, 649–659. 10.1016/j.cmet.2013.09.008 24120943 PMC3980968

[B259] Wu J. W. Hussaini S. A. Bastille I. M. Rodriguez G. A. Mrejeru A. Rilett K.. (2016). Neuronal activity enhances tau propagation and tau pathology *in vivo*. Nat. Neurosci. 19, 1085–1092. 10.1038/nn.4328 27322420 PMC4961585

[B260] Xie L. Kang H. Xu Q. Chen M. J. Liao Y. Thiyagarajan M.. (2013). Sleep drives metabolite clearance from the adult brain. Science 342, 373–377. 10.1126/science.1241224 24136970 PMC3880190

[B262] Xu W. Wang H. Wan Y. Tan C. Li J. Tan L.. (2017). Alcohol consumption and dementia risk: a dose-response meta-analysis of prospective studies. Eur. J. Epidemiol. 32, 31–42. 10.1007/s10654-017-0225-3 28097521

[B261] Xu J. Wei C. Xu C. Bennett M. C. Zhang G. Li F.. (2007). Rifampicin protects PC12 cells against MPP+-induced apoptosis and inhibits the expression of an α-synuclein multimer. Brain Res. 1139, 220–225. 10.1016/j.brainres.2006.12.074 17280646

[B263] Yamamoto K. Tanei Z. I. Hashimoto T. Wakabayashi T. Okuno H. Naka Y.. (2015). Chronic optogenetic activation augments Aβ pathology in a mouse model of Alzheimer disease. Cell Rep. 11, 859–865. 10.1016/j.celrep.2015.04.017 25937280

[B265] Yan X.-X. Cai Y. Shelton J. Deng S.-H. Luo X.-G. Oddo S.. (2012). Chronic temporal lobe epilepsy is associated with enhanced Alzheimer-like neuropathology in 3×Tg-AD mice. PLoS One 7:e48782. 10.1371/journal.pone.0048782 23155407 PMC3498246

[B264] Yan L. M. Velkova A. Kapurniotu A. (2014). Molecular characterization of the hetero-assembly of β-amyloid peptide with islet amyloid polypeptide. Curr. Pharm. Des. 20, 1182–1191. 10.2174/13816128113199990064 23713771

[B266] Yuede C. M. Zimmerman S. D. Dong H. Kling M. J. Bero A. W. Holtzman D. M.. (2009). Effects of voluntary and forced exercise on plaque deposition, hippocampal volume, and behavior in the Tg2576 mouse model of Alzheimer’s disease. Neurobiol. Dis. 35, 426–432. 10.1016/j.nbd.2009.06.002 19524672 PMC2745233

[B267] Zemlan F. P. Rosenberg W. S. Luebbe P. A. Campbell T. A. Dean G. E. Weiner N. E.. (1999). Quantification of axonal damage in traumatic brain injury: affinity purification and characterization of cerebrospinal fluid tau proteins. J. Neurochem. 72, 741–750. 10.1046/j.1471-4159.1999.0720741.x 9930748

[B268] Zetterberg H. Mörtberg E. Song L. Chang L. Provuncher G. K. Patel P. P.. (2011). Hypoxia due to cardiac arrest induces a time-dependent increase in serum amyloid β levels in humans. PLoS One 6:e28263. 10.1371/journal.pone.0028263 22194817 PMC3237426

[B269] Zhou S. Zhou R. Zhong T. Li R. Tan J. Zhou H. (2014). Association of smoking and alcohol drinking with dementia risk among elderly men in China. Curr. Alzheimer Res. 11, 899–907. 10.2174/1567205011666141001123356 25274108 PMC4428477

[B270] Zissimopoulos J. M. Barthold D. Brinton R. D. Joyce G. (2017). Sex and race differences in the association between statin use and the incidence of Alzheimer disease. JAMA Neurol. 74, 225–232. 10.1001/jamaneurol.2016.3783 27942728 PMC5646357

